# Supplementary material for: Inward rectifier potassium channels interact with calcium channels to promote robust and physiological bistability
Source: PLoS Comput Biol. 2026 Jul 28;22(7):e1014549. doi: 10.1371/journal.pcbi.1014549 (PMC13441080; doi:10.1371/journal.pcbi.1014549)
Supplement: Supplementary file 1 [file pcbi.1014549.s001.pdf]

Supporting information

S1 Appendix. Initially, the minimal model did not show bistability. The minimal model used in this study did not exhibit bistability initially, i.e. when only voltage-gated sodium channels, delayed-rectifier potassium channels, and a leak current were considered (S1 Fig A).

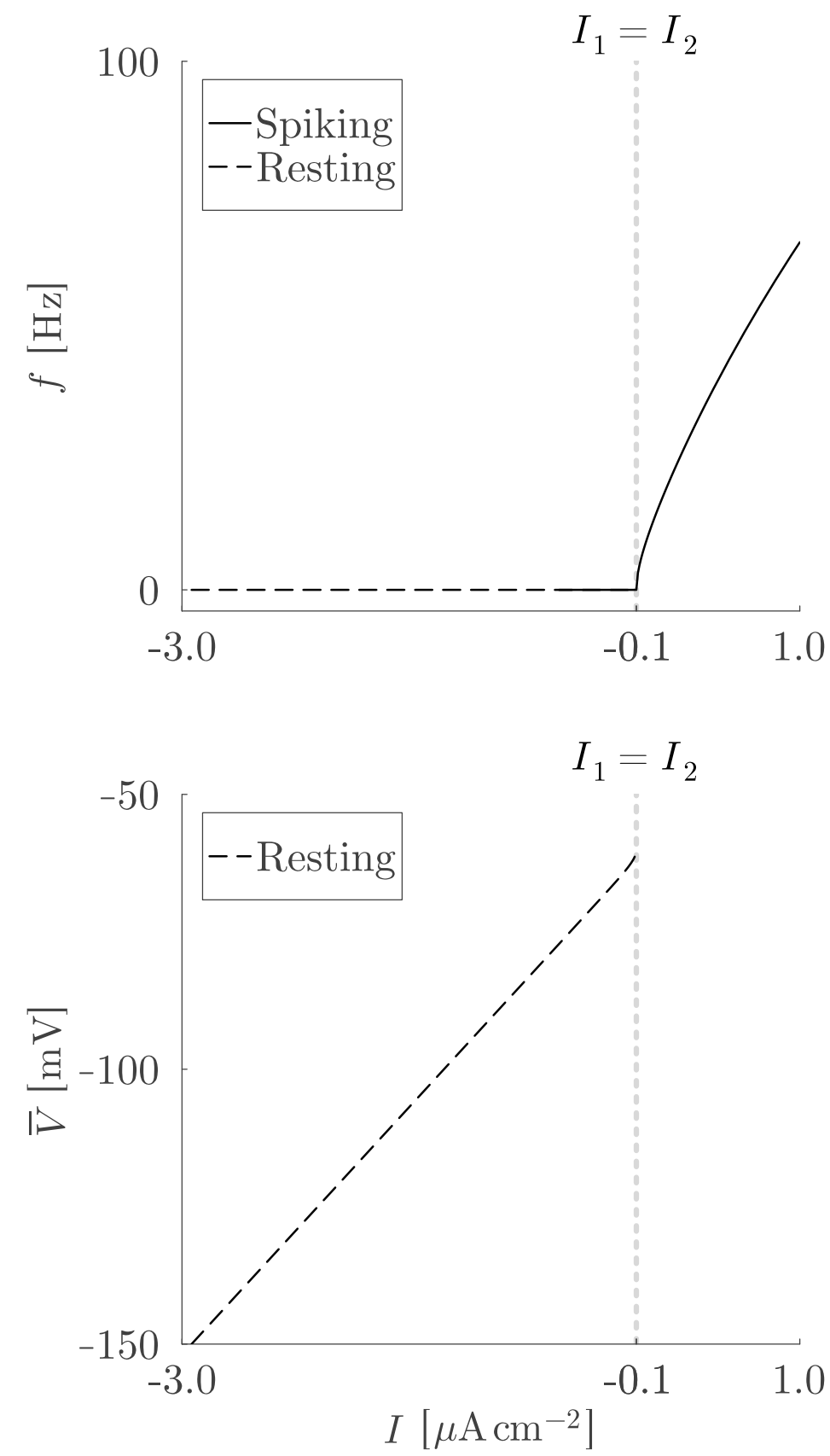

S1 Fig A. Without CaL channels, the model initially did not show bitability. The superimposition of the fI curves computed from resting and spiking, respectively, did not show the bistability window (top), that is, spiking appeared when resting disappeared as the applied current increased. The resting equilibria were progressively depolarized as the current increased (bottom).
