# Supplementary material for: Inward rectifier potassium channels interact with calcium channels to promote robust and physiological bistability
Source: PLoS Comput Biol. 2026 Jul 28;22(7):e1014549. doi: 10.1371/journal.pcbi.1014549 (PMC13441080; doi:10.1371/journal.pcbi.1014549)
Supplement: Supplementary file 2 [file pcbi.1014549.s002.pdf]

Supporting information

S2 Appendix. The increase and decrease of the bistability window size provided by Kir and KM channels, respectively, are still observed for several models of the CaL current. In [1], the decision to model the L-type calcium current as proportional to m_{CaL}^2 or to m_{CaL} was slightly unclear. This decision was investigated here to ensure that these choices did not impact our results. In the same view, the impact of incorporating a Goldman–Hodgkin–Katz formalism was explored to determine whether the neglect of the calcium concentration dependency in I_{CaL} would alter the conclusions drawn here. For that purpose, we modified the model of I_{CaL} and repeated the experiment in Fig 3A and C. The results of this experiment can be found in S2 Fig A and S2 Fig B, where $GHK(V, \left[\mathrm{Ca}^{2+}\right]_i)$ denotes the Goldman–Hodgkin–Katz formalism described in the Methods section. These results showed that the increase and decrease of the bistability window size provided Kir and KM channels (respectively) in combination with CaL channels are still observed for each model of I_{CaL} considered.

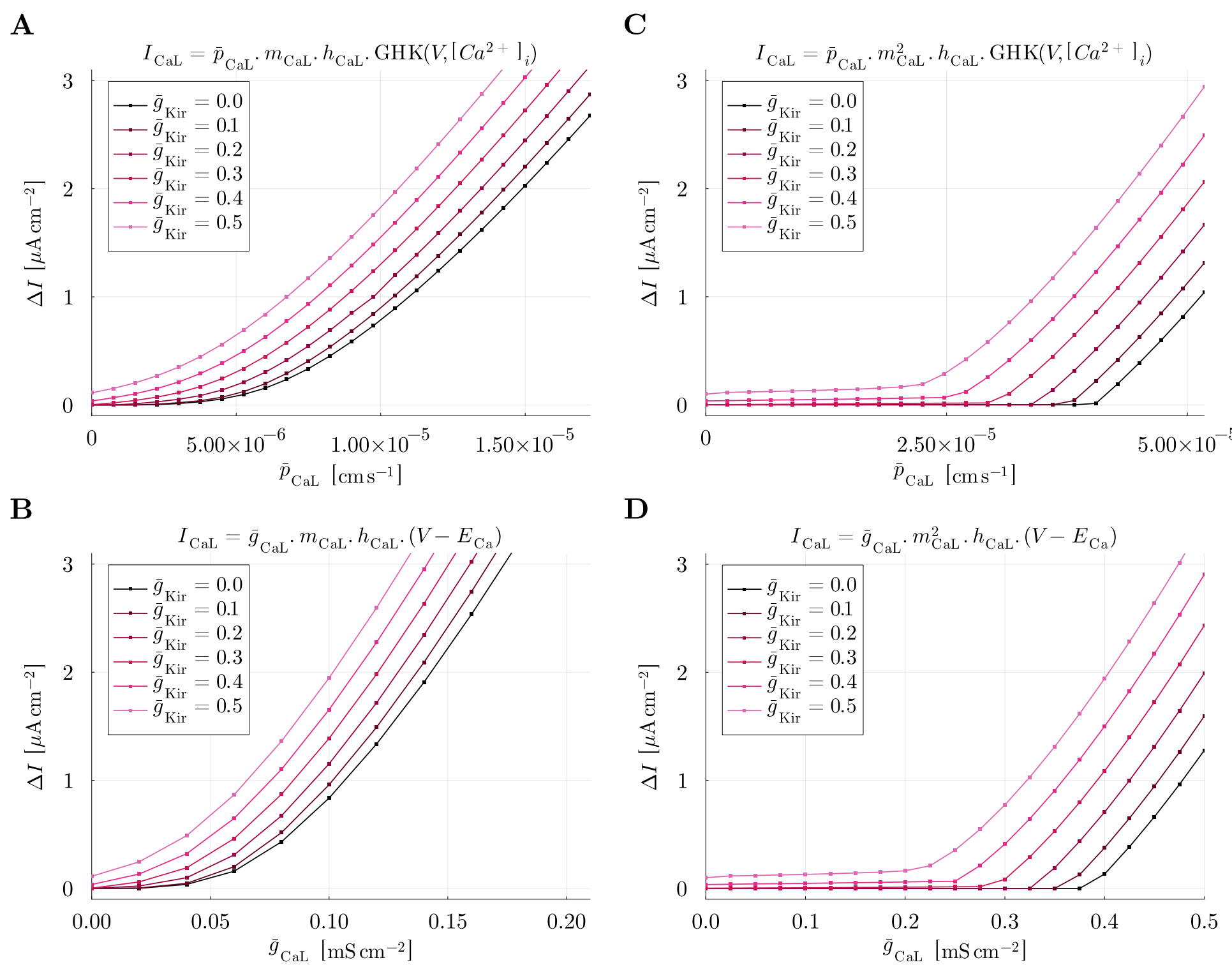

S2 Fig A. The increase in bistability window size induced by Kir channels is observed for q_{CaL} of 1 and 2, and whether or not calcium-dependent interactions are considered. A: Evolution of the bistability window size ΔI for an increasing permeability $\bar{p}_{\mathrm{CaL}}$ and several values of the maximal conductance $\bar{g}_{\mathrm{Kir}}$, for a CaL current embedding a calcium-dependency and $q_{\mathrm{CaL}} = 1$. B: Same as A for a CaL current embedding a calcium-dependency and $q_{\mathrm{CaL}} = 2$. C: Same as A for a CaL current, neglecting a calcium-dependency and $q_{\mathrm{CaL}} = 1$. D: Same as A for a CaL current neglecting a calcium-dependency and $q_{\mathrm{CaL}} = 2$.

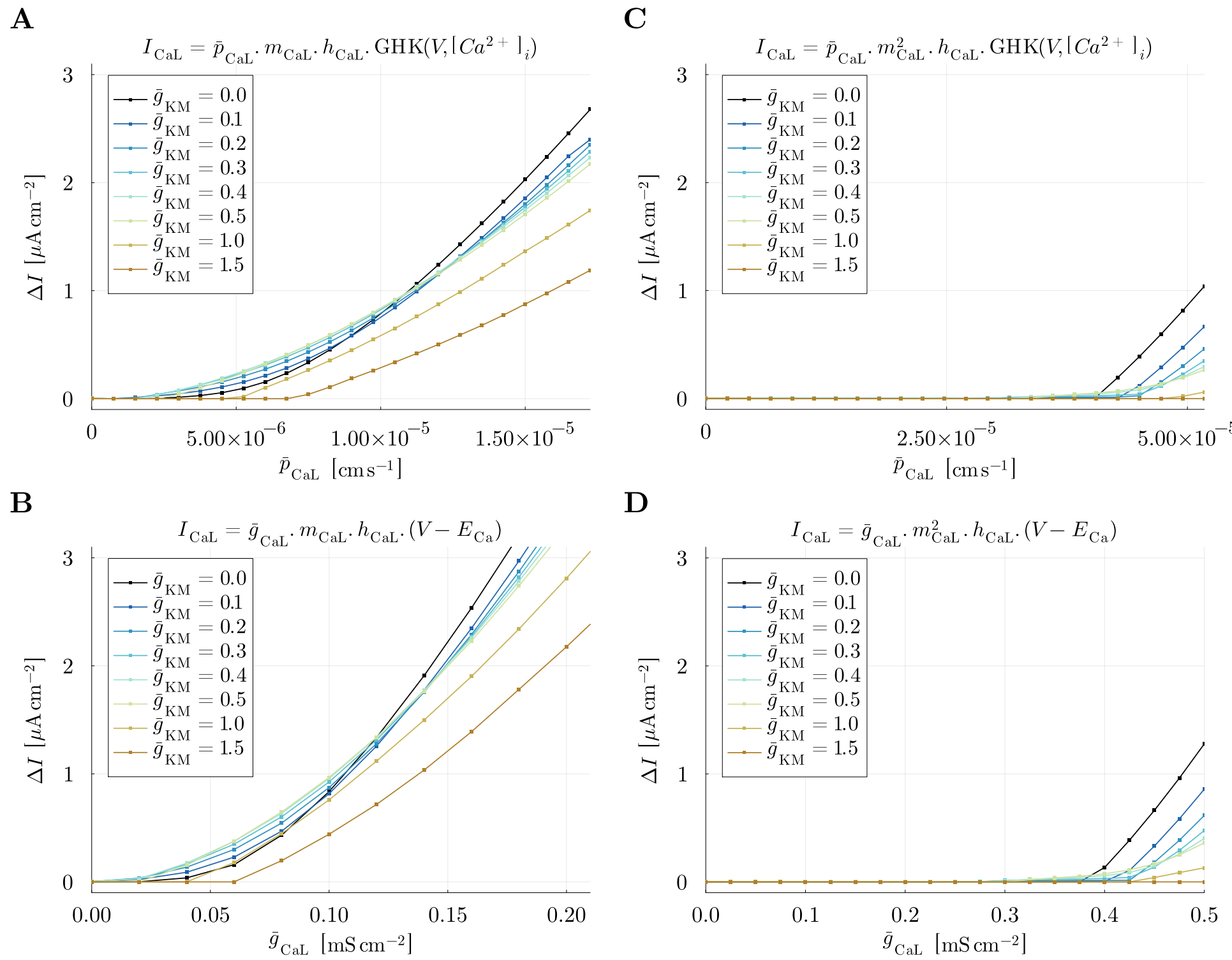

S2 Fig B. The reduction in the bistability window size induced by KM channels is observed for q_{CaL} of 1 and 2, and whether or not calcium-dependent interactions are considered. A: Evolution of the bistability window size ΔI for an increasing permeability $\bar{p}_{\mathrm{CaL}}$ and several values of the maximal conductance $\bar{g}_{\mathrm{KM}}$, for a CaL current embedding a calcium-dependency and $q_{\mathrm{CaL}} = 1$. B: Same as A for a CaL current embedding a calcium-dependency and $q_{\mathrm{CaL}} = 2$. C: Same as A for a CaL current neglecting a calcium-dependency and $q_{\mathrm{CaL}} = 1$. D: Same as A for a CaL current neglecting a calcium-dependency and $q_{\mathrm{CaL}} = 2$.

References

1. Le Franc Y, Le Masson G. Multiple Firing Patterns in Deep Dorsal Horn Neurons of the Spinal Cord: Computational Analysis of Mechanisms and Functional Implications. Journal of Neurophysiology. 2010;104(4):1978–1996. doi:10.1152/jn.00919.2009.
