# Supplementary material for: Inward rectifier potassium channels interact with calcium channels to promote robust and physiological bistability
Source: PLoS Comput Biol. 2026 Jul 28;22(7):e1014549. doi: 10.1371/journal.pcbi.1014549 (PMC13441080; doi:10.1371/journal.pcbi.1014549)
Supplement: Supplementary file 3 [file pcbi.1014549.s003.pdf]

Supporting information

S3 Appendix. Both Kir and KM channels increased the narrow bistability window resulting from a $\bar{p}_{\mathrm{CaL}}$ value that was halved compared to that used in Fig 4. We performed a similar analysis to what was made in Fig 4 with half the value used for $\bar{p}_{\mathrm{CaL}}$, which created a bistability window size of $\Delta I_0 = 0.34\,\mu\mathrm{A/cm}^2$. Increasing both Kir and KM conductances between 0 and $0.3\,\mathrm{mS/cm}^2$ showed that both Kir and KM channels may increase the bistability window size, if initially lower that what was considered n Fig 4. The largest increase in the bistability window size observed as $\bar{g}_{\mathrm{KM}}$ was increased to $0.3\,\mathrm{mS/cm}^2$ was of $0.14\,\mu\mathrm{A\,cm}^{-1}$, obtained with $\bar{g}_{\mathrm{Kir}} = 0$ (S3 Fig AA). Similar to the results shown in Fig 4, Kir and KM promoted different ranges of resting equilibria (S3 Fig AB and C).

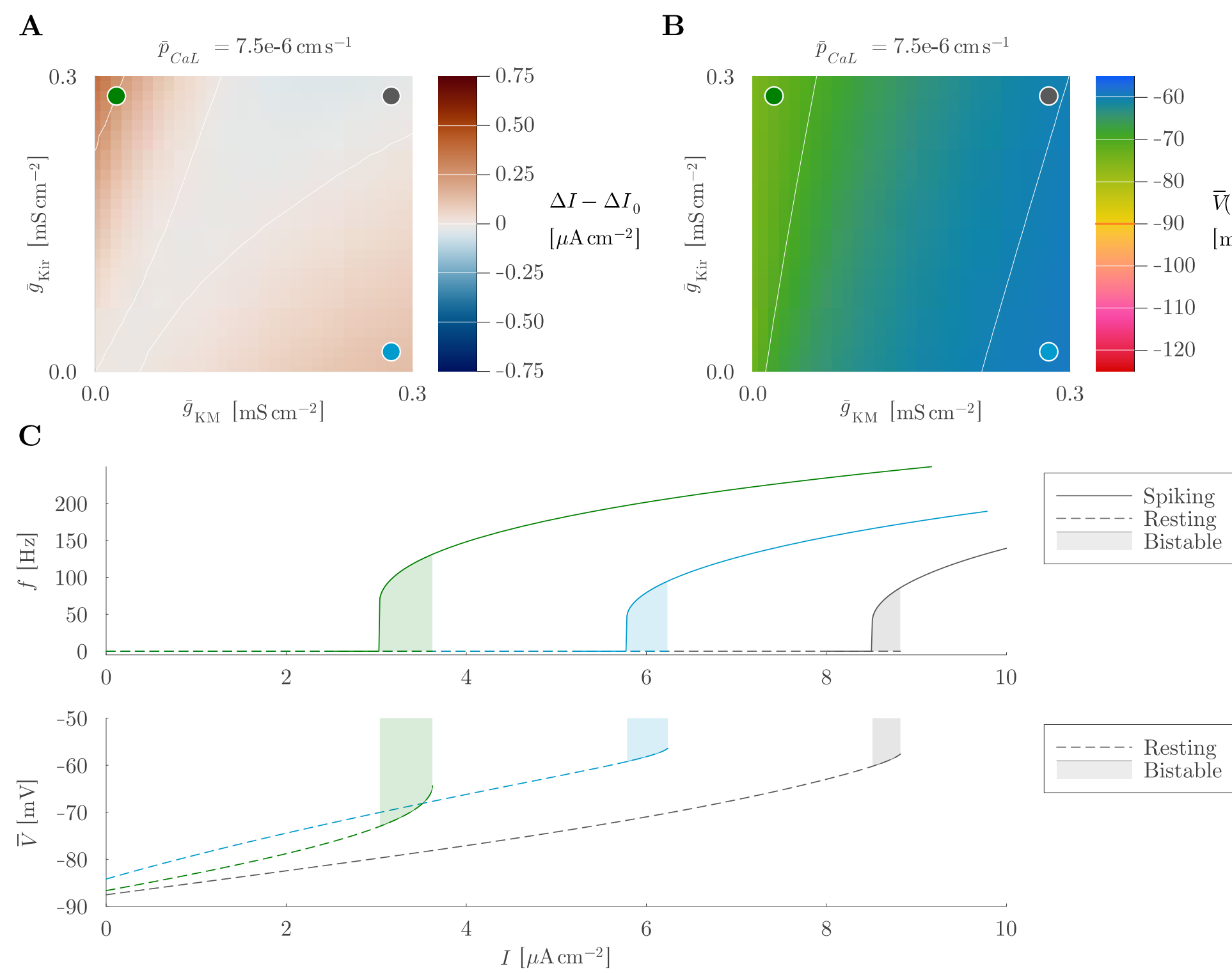

S3 Fig A. With an initial bistability window size of $\Delta I_0 = 0.34\,\mu\mathrm{A/cm}^2$, Kir and KM channels increase separately the bistability window size, but promote distinct ranges of resting equilibria. A: Heatmap of the absolute change in bistability window size (ΔI) from the bistability window size resulting from $\bar{p}_{\mathrm{CaL}} = 7.5 \times 10^{-6}\,\mathrm{cm\,s}^{-1}$ and $\bar{g}_{\mathrm{KM}} = \bar{g}_{\mathrm{Kir}} = 0$ (noted ΔI_0, equal to $0.34\,\mu\mathrm{A/cm}^2$), depending on the pair of conductances $(\bar{g}_{\mathrm{KM}}, \bar{g}_{\mathrm{Kir}})$ used. B: Heatmap of the resting equilibrium at the lower bound of the bistability window ($\overline{V}(I_1)$), depending on the pair of conductances $(\bar{g}_{\mathrm{KM}}, \bar{g}_{\mathrm{Kir}})$ used. C: Superimposed fI curves from resting and spiking (top) and resting membrane potentials observed for a range of applied current, both displaying the bistability window, corresponding to the 3 sets of parameters marked in A and B. The white lines in A and B represent contour lines for each heatmap.
