# Supplementary material for: Inward rectifier potassium channels interact with calcium channels to promote robust and physiological bistability
Source: PLoS Comput Biol. 2026 Jul 28;22(7):e1014549. doi: 10.1371/journal.pcbi.1014549 (PMC13441080; doi:10.1371/journal.pcbi.1014549)
Supplement: Supplementary file 4 [file pcbi.1014549.s004.pdf]

Supporting information

S4 Appendix. Replacing Kir by KM channels in the complete conductance-based model shows that KM channels decrease the bistability window size. We performed the same experiments as in Fig 5 but replaced the Kir channels with KM channels. For low $\bar{p}_{\mathrm{CaL}}$, there was no bistability observed in the pulse responses (S4 Fig AA and B). Increasing $\bar{p}_{\mathrm{CaL}}$ allowed to observed bistability, for lower and higher values of $\bar{g}_{\mathrm{KM}}$ (S4 Fig AC and D). The superimposed fI curves confirmed the absence of bistability for low $\bar{p}_{\mathrm{CaL}}$, for the two values of $\bar{g}_{\mathrm{KM}}$ considered (S4 Fig AE, top). Increasing $\bar{p}_{\mathrm{CaL}}$ resulted in the superimposed fI curves displaying a bistability window for the two values of $\bar{g}_{\mathrm{KM}}$ (S4 Fig AF, top). Increasing $\bar{g}_{\mathrm{KM}}$ starting from either of the values of $\bar{p}_{\mathrm{CaL}}$ used increased the resting equilibria observed (S4 Fig AE and F, bottom). Using a range of $\bar{p}_{\mathrm{CaL}}$ and $\bar{g}_{\mathrm{KM}}$ showed that KM channels decrease slightly the size of the bistability window for $\bar{p}_{\mathrm{CaL}} \approx 1.5 \times 10^{-5}\,\mathrm{cm\,s^{-1}}$ (S4 Fig AG), which is similar to the results obtained with the minimal model in Fig 3A. For $\bar{p}_{\mathrm{CaL}}$ above $2 \times 10^{-5}\,\mathrm{cm\,s^{-1}}$, the reduction is ΔI is stronger as $\bar{g}_{\mathrm{KM}}$ increases. Whereas CaL channels showed a monotonic increase in ΔI for $\bar{g}_{\mathrm{KM}} = 0$, increasing $\bar{g}_{\mathrm{KM}}$ broke this monotonicity. In fact, the increase of ΔI for $\bar{p}_{\mathrm{CaL}} \approx 1.5 \times 10^{-5}\,\mathrm{cm\,s^{-1}}$ dropped as $\bar{p}_{\mathrm{CaL}}$ was increased further. KM channels also increased I_1 (S4 Fig AH) and $\overline{V}(I_1)$ (S4 Fig AI).

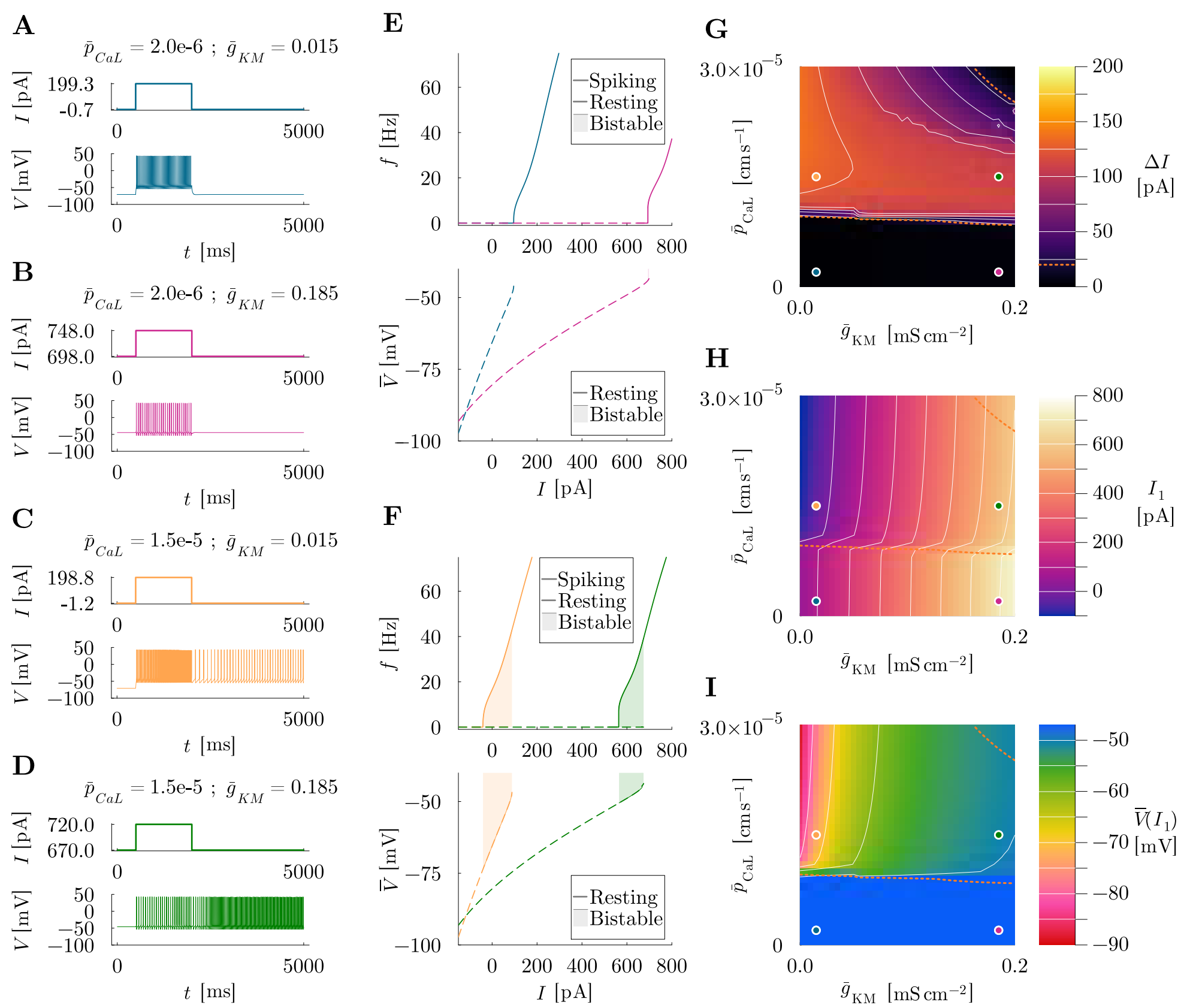

S4 Fig A. In a more complete two-compartment model of deep projection neurons, replacing Kir by KM channels shows that KM channels slightly decrease ΔI, or even decrease it to 0 pA. A: Response of the two-compartment model with a low $\bar{p}_{\mathrm{CaL}}$ and a low $\bar{g}_{\mathrm{KM}}$ initially at −65 mV to a pulse of current of 200 pA. B: Same as A, but with a $\bar{g}_{\mathrm{KM}}$ increased to 0.185 mS/cm^2 and a 50 pA pulse of current. C: Same as A, but with a $\bar{p}_{\mathrm{CaL}}$ increased to 1.5×10^{-5} cm s^{-1}. D: Same as C with a $\bar{p}_{\mathrm{CaL}}$ increased to 1.5×10^{-5} cm s^{-1}. E: Superimposed fI curves from resting and spiking (top) and resting membrane potentials observed for a range of applied current (bottom), both displaying the bistability window (if existing), corresponding to the 2 sets of parameters used in A and C. F: Same as E, but for the 2 sets of parameters used in B and D. G: Heatmap of the bistability window size (ΔI) depending on the pair of conductances ($\bar{g}_{\mathrm{KM}}, \bar{p}_{\mathrm{CaL}}$) used. H: Heatmap of the bistability window lower limit (I_1) obtained for each pair of conductances. I: Heatmap of the resting equilibrium observed at bistability window lower limit ($\overline{V}(I_1)$) for each pair of conductances. Each heatmap contained 41×31 data points. The white lines in G, H and I represent contour lines for each heatmap. The red dashed line marks $\Delta I = 20$ pA.
