# Supplementary material for: Inward rectifier potassium channels interact with calcium channels to promote robust and physiological bistability
Source: PLoS Comput Biol. 2026 Jul 28;22(7):e1014549. doi: 10.1371/journal.pcbi.1014549 (PMC13441080; doi:10.1371/journal.pcbi.1014549)
Supplement: Supplementary file 5 [file pcbi.1014549.s005.pdf]

Supporting information

S5 Appendix. Resting states are more robust to noise than spiking states at I_1 considering whether KM or Kir channels are considered in combination with CaL channels. Here, we performed the same experiments as in Fig 6 but with I_1 instead of the central current of the bistability window ($i.e. \frac{I_1+I_2}{2}$) considering either KM or Kir channels in combination with CaL channels (S5 Fig A). This experiment quantifies the robustness to noise of resting and spiking for $\bar{g}_{\mathrm{KM}}$-bistability and $\bar{g}_{\mathrm{Kir}}$-bistability. Compared to Fig 6, the resting states were more robust at I_1 than at $\frac{I_1+I_2}{2}$, but the spiking states were more robust at $\frac{I_1+I_2}{2}$ than at I_1, with both types of bistability. This is consistent with a higher(resp. lower) reachability of the resting(resp. spiking) state at I_1 than at $\frac{I_1+I_2}{2}$ as observed in Fig 7. For both $\bar{g}_{\mathrm{KM}}$-bistability and for $\bar{g}_{\mathrm{Kir}}$-bistability, the resting state in I_1 was more robust than the spiking state. This may be due to the higher reachability of the resting state than of the spiking state in I_1, as observed in Fig 7. Finally, these results showed that for both in I_1 and in $\frac{I_1+I_2}{2}$, resting with $\bar{g}_{\mathrm{Kir}}$-bistability was more robust than resting with $\bar{g}_{\mathrm{KM}}$-bistability, consistently with a higher reachability throughout the bistability window of the resting state with $\bar{g}_{\mathrm{Kir}}$-bistability compared to that with $\bar{g}_{\mathrm{KM}}$-bistability.

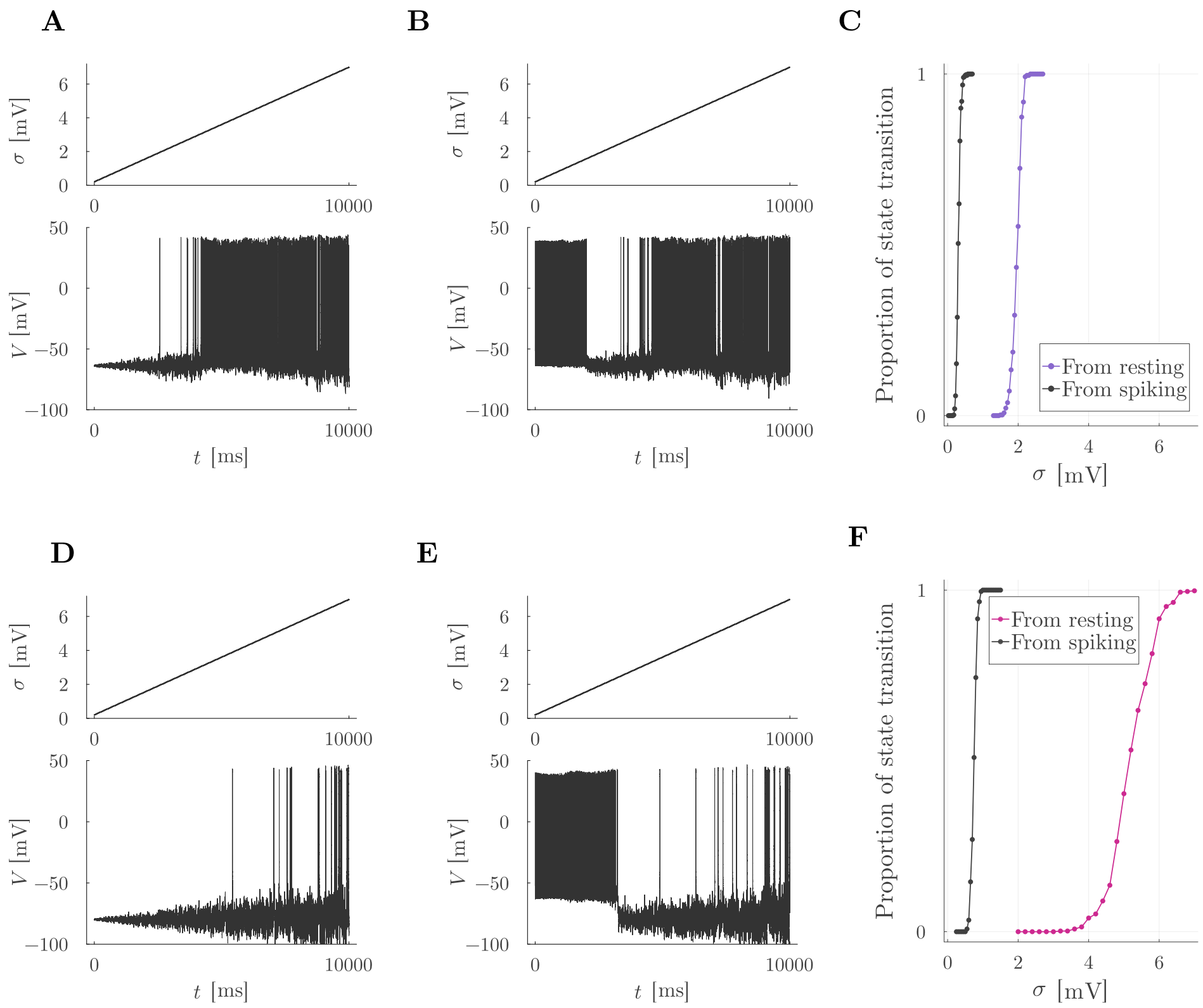

S5 Fig A. At the beginning of the bistability window, resting states are more robust to noise than spiking states considering whether KM or Kir channels are considered in combination with CaL channels. A: Response of the model including I_{KM} to I_1 with an additional white noise of increasing standard deviation σ, when initially resting. B: Same as A, when initially spiking. C: Proportion of trials that failed to maintain their initial behavior (resting or spiking) and transitioned to spiking or resting, respectively, as a function of the value of constant standard deviation σ used to create white noise, with the model including I_{KM}.
D: Same as A, but with the model including I_{Kir} instead of I_{KM}. E: Same as A, but with the model including I_{Kir} instead of I_{KM}, and when initially spiking. F: Same as C, but with the model including I_{Kir} instead of I_{KM}. The bistability window size in A, B, and C, is similar to those of D, E, and F (resulting from the addition of I_{Kir}), such that the same value of maximal conductance was used for $\bar{g}_{\mathrm{KM}}$ (in A, B, and C) and for $\bar{g}_{\mathrm{Kir}}$ (in D, E, and F), but with a higher $\bar{p}_{\mathrm{CaL}}$ in A, B, and C compared to D, E, and F.
