# Supplementary material for: Inward rectifier potassium channels interact with calcium channels to promote robust and physiological bistability
Source: PLoS Comput Biol. 2026 Jul 28;22(7):e1014549. doi: 10.1371/journal.pcbi.1014549 (PMC13441080; doi:10.1371/journal.pcbi.1014549)
Supplement: Supplementary file 6 [file pcbi.1014549.s006.pdf]

Supporting information

S6 Appendix. The other faster L-type calcium current described by [1] has a reduced impact on bistability. [1] described and modeled two types of L-type calcium channels. In this study, only the slower type of L-type calcium channels was considered, but whether the faster type was also able to create bistability was unclear. To explore its effect on bistability, we added the ionic current arising from the faster type of L-type calcium channels ($I_{\text{CaL,f}}$) to the model. The fast L-type calcium current, embedding a calcium-dependency, was modeled using a Goldman–Hodgkin–Katz formalism [2] such that:

$$I_{\text{CaL,f}} = \bar{p}_{\text{CaL,f}}\, m_{\text{CaL,f}}^2\, h_{\text{CaL,f}} \text{GHK}(V, \left[\text{Ca}^{2+}\right]_i), \tag{1}$$

$$\text{with GHK}(V, \left[\text{Ca}^{2+}\right]_i) = 10^{-3} \cdot 2F \cdot \left(\left[\text{Ca}^{2+}\right]_i \cdot \frac{-w}{e^{-w}-1} - \left[\text{Ca}^{2+}\right]_o \cdot \frac{w}{e^{w}-1} \right), \tag{2}$$

$$\text{and } w = 10^{-3} \cdot V \cdot \frac{2F}{RT}. \tag{3}$$

Each variable had the same value and meaning as described in the Methods section. The steady-state curves of the activation $m_{\text{CaL,f}}$ and inactivation $h_{\text{CaL,f}}$ can be found in S6 Table A. The evolution of the intracellular calcium concentration was modified to take $I_{\text{CaL,f}}$ into account as part of the total calcium current flowing through the membrane, such that:

$$\frac{d\left[\text{Ca}^{2+}\right]_i}{dt} = -10^7 \cdot \frac{(I_{\text{CaL}} + I_{\text{CaL,f}})}{10^3} \cdot \frac{1}{2Fd} + \frac{\left(\left[\text{Ca}^{2+}\right]_{i,0} - \left[\text{Ca}^{2+}\right]_i\right)}{\tau_{\text{Ca}}}. \tag{4}$$

To assess the effect of $I_{\text{CaL,f}}$ on bistability, the experiment realized in Fig 3A and C was repeated with two fixed value of permeability $\bar{p}_{\text{CaL,f}}$ or 0 and $1.5 \times 10^{-4}\,\text{cm}\,\text{s}^{-1}$. As observed in S6 Fig A, the faster L-type calcium current did not create bistability itself; rather, it appeared to widen the bistability window engendered by the slower L-type calcium current.

S6 Table A. Steady-state activations and inactivation used to model I_{CaLf}.

x_{ion}	$x_{\text{ion},\infty}(V)$	$\tau_{x_{\text{ion}}}(V)$
m_{CaLf}	$\frac{1}{1+\exp(-(V+17.5)/4.3)}$	$\frac{0.5}{\frac{0.1(-V-40)}{\exp(0.1(-V-40))-1+4\exp((-V-65)/18)}}$
h_{CaLf}	$\frac{1}{1+\exp((V+14)/4.03)}$	1500

References

1. Le Franc Y, Le Masson G. Multiple Firing Patterns in Deep Dorsal Horn Neurons of the Spinal Cord: Computational Analysis of Mechanisms and Functional Implications. Journal of Neurophysiology. 2010;104(4):1978–1996. doi:10.1152/jn.00919.2009.

2. Hodgkin AL, Katz B. The effect of sodium ions on the electrical activity of the giant axon of the squid. The Journal of Physiology. 1949;108(1):37–77. doi:10.1113/jphysiol.1949.sp004310.

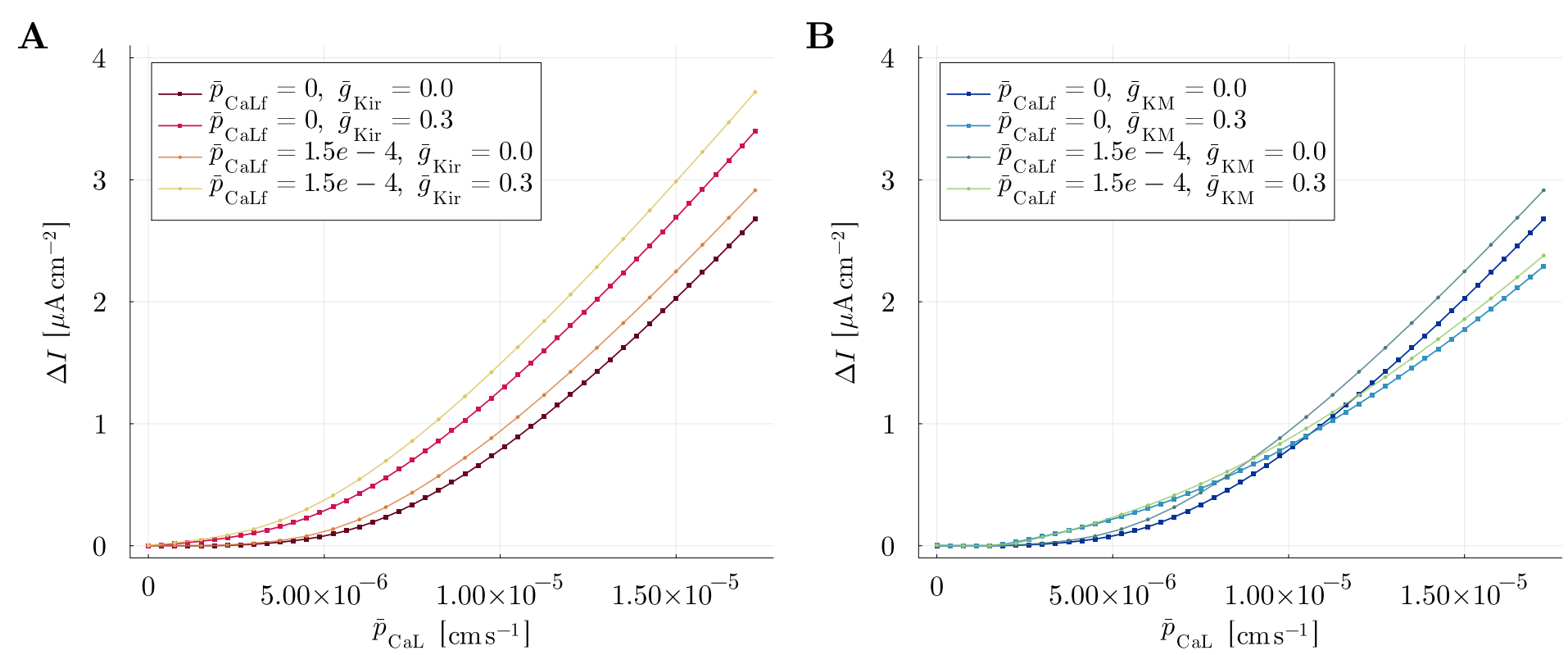

S6 Fig A. The other L-type calcium current ($I_{\mathrm{CaL,f}}$) described by [1] has a reduced impact on bistability compared to the L-type calcium current considered in this work ($I_{\mathrm{CaL,s}}$), whether or not the Kir or KM current were considered. A: Evolution of the bistability window size ΔI for an increasing permeability $\bar{p}_{\mathrm{CaL,s}}$, two values of the maximal conductance $\bar{g}_{\mathrm{Kir}}$, and two values of permeability $\bar{p}_{\mathrm{CaL,f}}$ of the other type of L-type calcium channels. B: Same as A with several values of $\bar{g}_{\mathrm{KM}}$ instead of $\bar{g}_{\mathrm{Kir}}$.
