# Supplementary material for: Inward rectifier potassium channels interact with calcium channels to promote robust and physiological bistability
Source: PLoS Comput Biol. 2026 Jul 28;22(7):e1014549. doi: 10.1371/journal.pcbi.1014549 (PMC13441080; doi:10.1371/journal.pcbi.1014549)
Supplement: Supplementary file 7 [file pcbi.1014549.s007.pdf]

Supporting information

S7 Appendix. The reduction in the bistability window size is still provided by KM channels opening at a higher membrane potential. In this study, the steady-state activation curve of KM channels was modified to allow the comparison with Kir channels within the same range of potentials. However, to demonstrate that the decrease in the bistability window size caused by the addition of KM channels was still present without this modification, the experiment realized for Fig 3A and C was repeated with the unmodified steady-state activation curve of KM channels, which can be found in S7 Table A. Those results can be found in S7 Fig A.

S7 Table A. Steady-state activation used to model $I_{\mathrm{KM'}}$.

x_{ion}	$x_{\mathrm{ion},\infty}(V)$	$\tau_{x_{\mathrm{ion}}}(V)$
$m_{\mathrm{KM'}}$	$\frac{1}{1+\exp((-V-26.7)/12.6)}$	103

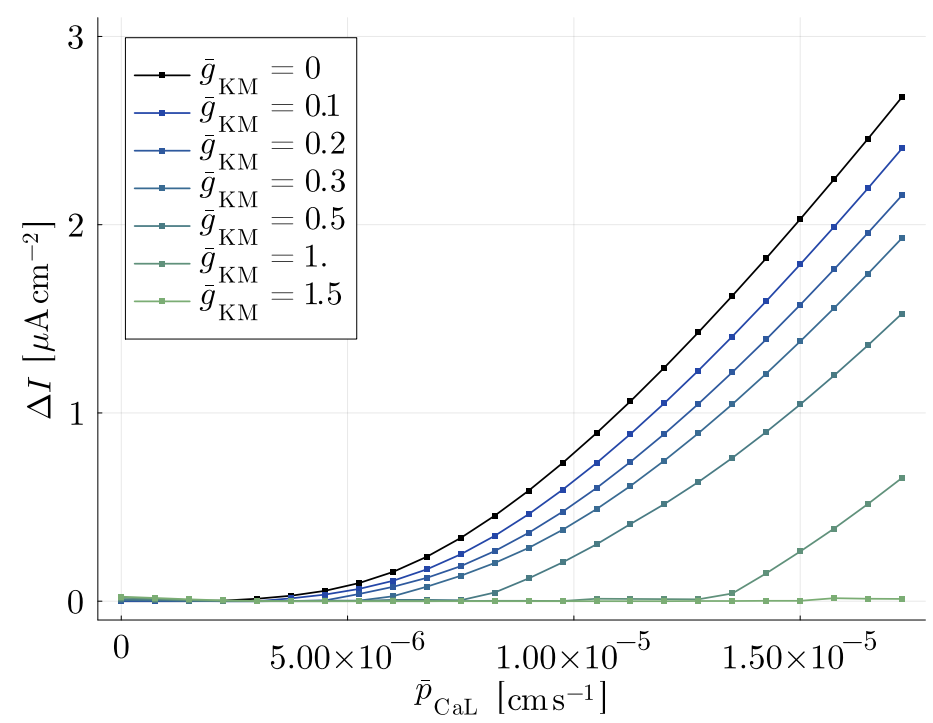

S7 Fig A. The reduction in the bistability window size induced by KM channels is robust to a change in the KM channel steady-state activation to $m_{\mathrm{KM},\infty} = \frac{1}{1+\exp((-V-26.7)/12.6)}$. Evolution of the bistability window size ΔI for an increasing permeability $\bar{p}_{\mathrm{CaL}}$, and several values of the maximal conductance $\bar{g}_{\mathrm{KM}}$.
