# Supplementary material for: Inward rectifier potassium channels interact with calcium channels to promote robust and physiological bistability
Source: PLoS Comput Biol. 2026 Jul 28;22(7):e1014549. doi: 10.1371/journal.pcbi.1014549 (PMC13441080; doi:10.1371/journal.pcbi.1014549)
Supplement: Supplementary file 8 [file pcbi.1014549.s008.pdf]

Summary

In the study “Inward rectifier potassium channels interact with calcium channels to promote robust and physiological bistability,” the authors investigate a potentially important mechanism underlying the sensitization of nociceptive projection neurons in the spinal cord following intense pain stimuli. Using single-compartment models, they explore how L-type calcium channels and potassium channels (Kir vs. KM) may contribute to bistability—a phenomenon that could support persistent neuronal sensitization. While the topic is compelling and relevant, the study in its current form has several major limitations.

Major 1: Lack of Comparison to Empirical Data.

The authors do not compare their model results to electrophysiological data from the neuron type they investigate. This raises concerns about the physiological relevance of their findings. For instance, the firing rates reported in Figure 1C reach 100–200 Hz, whereas Monteiro et al. — cited in the manuscript — report typical firing rates of 10–20 Hz in rat dorsal horn neurons. This discrepancy calls into question whether the model reflects realistic neuronal dynamics.

Furthermore, the authors frequently distinguish between “physiological” and “non-physiological” bistability, but this distinction is not clearly defined or grounded in empirical observations. The study uses the “bistability window” — the range of input currents for which the neuron’s output depends on its initial condition — as a measure to quantify bistability. However, the manuscript does not clarify what range of bistability windows would be considered physiological.

To strengthen the study, the authors should define their use of the term “physiological”, provide quantifications of their models responses (e.g., firing rates, bistability windows, AP amplitudes, AP width, amplitudes of the plateau potential), and compare them to empirical data from dorsal horn neurons.

Major 2: Limited Exploration of Parameter Space

Throughout most of the study, the authors use a fixed base model to which either CaL + Kir or CaL + KM channels are added. However, it remains unclear to what extent the results depend on the specific expression levels of the other ion channels included in the model, and whether the findings would fundamentally change in the presence of additional conductances that are expressed in dorsal horn neurons. While the use of a minimal model to explore how bistability can arise in principle is understandable (and I consider in particular their bifurcation analysis as a strength of their study), the authors should also construct a model that reproduces the characteristic electrophysiological properties of rat

dorsal horn neurons and verify that their main conclusions generalize to this more realistic scenario.

Major 3: Inconsistent and Ambiguous Terminology

Terminology varies throughout the manuscript, and several key terms are not clearly defined. This inconsistency creates confusion and hinders interpretation of the results.

For example, the following phrases appear in different sections of the manuscript and seem to describe the same concept:

- "trade-off between bistability and the plausibility of resting states" (Abstract)
- "trade-off between bistability and the plausibility of resting potentials" (Author summary)
- "trade-off between bistability robustness and resting potential adjustment" (line 27)
- "trade-off between the bistability window size and the plausibility of the resting potentials" (line 141)
- "trade-off exists between the bistability window size and the resting potential adjustment" (line 146)
- "trade-off observed between the bistability window size and the adjustment of its equilibrium potentials" (line 355)

Do all of these refer to the same? The manuscript should consistently use a single term or clearly explain the distinctions. Additionally, does the use of "resting potential" and "equilibrium potential" imply that the authors treat them interchangeably? If so, this deviates from standard definitions — typically, *resting potential* refers to the steady-state membrane potential in the absence of stimulation , while *equilibrium potential* refers to the Nernst potential of a particular ion species. Fig 3 reports resting potentials far below the reversal potential of potassium channels, suggesting that the authors definition of these terms indeed deviates from the commonly used definition. I assume the authors refer to the voltage observed at current I_1?

Similar concerns apply to other vague or inconsistently used terms, including:

- "high level of bistability" (line 106)
- "weakened bistability" / "strengthened bistability" (line 52)
- "robust bistability"
- "reinforce bistability" (line 56)
- "classical potassium channels"

The authors should rewrite the manuscript to ensure each term is clearly defined before it is used for the first time and aligns with the definitions commonly used in the field, and that terminology is kept constant throughout the manuscript.

Minor 1: Missing Units on Figure Axes: Several figure panels don't have any units on their axis (e.g. Fig 3)

Minor 2: Several legends do not match the content of the figure. For instance, in Figure 8E, the legend refers to a "saddle node" line style that is not clearly visible in the plot. The line styles for "stable node" and "cycle extrema" appear nearly identical — possibly due to minimal differences in color shade? — making them hard to distinguish. Similar issues are present in other panels of Figure 8, as well as in Figure 9C–D. The authors should ensure that all line types, colors, and markers are distinct and clearly match the legend.

Minor 3: Use of Jargon. The manuscript uses technical terms from bifurcation theory (e.g., "saddle-node on invariant circle bifurcation," "saddle-homoclinic bifurcation") without explanation. While appropriate for readers with a mathematical modeling background, such terminology may limit accessibility for neuroscientists with primarily experimental or biological expertise.
