# Supplementary material for: Inward rectifier potassium channels interact with calcium channels to promote robust and physiological bistability
Source: PLoS Comput Biol. 2026 Jul 28;22(7):e1014549. doi: 10.1371/journal.pcbi.1014549 (PMC13441080; doi:10.1371/journal.pcbi.1014549)
Supplement: Supplementary file 10 [file pcbi.1014549.s010.pdf]

Review of “Inward rectifier potassium channels interact with calcium channels to promote robust and physiological bistability.”

The authors have significantly extended and improved the manuscript. Some of my previous concerns have been fully addressed, others remain, and a few new issues have emerged. With additional analysis, reframing to better match the data, and a concerted effort to improve clarity and conciseness, I believe the manuscript would be ready for acceptance.

Major 1: The authors binary presentation of “Kir promotes bistability, KM impairs bistability” is not supported by the data.

In Figure 3, the authors generate models with different parameter values for the maximal conductance of CaL + KM vs CaL + Kir channels and then quantify the bistability window size for each model. The figure shows that the bistability window size is largely insensitive to potassium channel expression and is dominated by CaL expression. KM has almost no effect (for high CaL values, it slightly reduces the window size, for low CaL values it may slightly increase it – but this hard to tell from the colormap, I suggest the authors include isopotential lines). Panel 3D, shows that Kir does have a stronger effect, which clearly matches what the authors say (the more Kir the larger the bistability window), but the effect of Kir still remains far below the effect of CaL.

The authors should highlight the parameter regions in in which their criteria for robust bistability and physiological membrane potentials are met. If I search e.g. for models with a window size > 2 and a V(I1) > - 70mV, it seems that there are many solutions for both the CaL+Kir and the CaL+KM channel.

This also calls into question the usefulness of the concept of a “trade-off between bistability and the plausibility of resting states”, as the authors clearly show that bistability with plausible membrane potentials can emerge with either channel. If the goal is simply to achieve bistability wihtin physiological constraints (not to *maximize* window size), then the trade-off dissolves — both channels can get you there, with window sizes that are about similar. The trade-off only matters if maximizing window size is biologically meaningful, which the authors haven't established.

The binary narrative of contrasting Kir with KM seems particularly extreme in the figure legend of Figure 2, Panel A, where the authors say “When adding KM […] the neuron response to a pulse of similar pulse amplitude as in Fig 1A shows that bistability […] is not observed anymore.” And for Panel D: “Replacing KM channels with Kir channels restores bistability between resting and spiking in the neuron response to a pulse of similar amplitude as A”. This is misleading. The authors chose to give a current stimulus that jumps from 0.9 to 3.9 and back for Panel A and a stimulus that jumps form -0.6 to 2.5 and back for Panel D. Importantly, 0.9 in Panel A is clearly below the bistability window for the KM model (I1 is 1.2 according to Panel 2B). In contrast, -0.6, chosen for the Kir model, is right at the border within its bistability

window. It is thus not surprising that the model returns to a stable resting state in Panel A. Had the authors replaced the stimulus protocols such that the base current for the Kir example is below I1 and the base current for the KM example is between I1 and I2, the figure would likely have demonstrate the opposite result.

Essentially, I would suggest to drop the binary framing, and instead focus on how the potassium channel kinetics can modulate bistability. The interpretation of modulation seems also adequate given Fig. 5, which shows the effect of Kir and CaL added to a more realistic model. Again, CaL shows up as the major determinant of bistability (with the bistability window size jumping up as soon as CaL reaches a threshold) whereas Kir conductance plays a minor role. I suggest to highlight models that meet the authors criteria also in this figure.

While I appreciate the effort of testing their prediction of the effect of Kir channels in a more realistic model, the authors did not perform a similar analysis combining KM with CaL in the more realistic model. Given that the simplified model showed viable parameter combinations for both Kir and KM, it seems likely that solutions meeting the authors' criteria would also exist for KM in the full model.

Major 2: The reasoning about the effect of the positive / negative differential conducantce on the bistability window size is hard to follow.

The section is difficult to follow, in parts due to inconsistent terminology and apparent figure reference errors.

Lines 481-491: "However, because they have both a region of positive and negative slope in 482 their steady-state current, their impact on the resting potentials is not straightforward. Below −60.1 mV, the leak current is the main driver defining the resting potentials. When Kir channels are added, the peak in the steady state Kir current found at −67 mV creates a local peak in the sum of the total ion current, that is in between −60.1 and −67 mV, and closer to the latter as the Kir conductance increases. Accordingly, as shown in Fig.3C (bottom part, for $\bar{p}_{CaL} \sim 0$), the increase in Kir channels conductance hyperpolarized the resting potential in I2, since in this region bistability was not observed I1 = I2. Respectively, when $\bar{p}_{CaL}$ was increased to 1.725×10^{-5} cm s^{-1}, bistability appeared, and Kir channels depolarized the resting potential in I1 (Fig.3C). In this scenario, I1 was lower than I2 "

The authors refer to Figure 3C – but this panel does not show Kir channel conductance. Do the authors mean Panel 3F? The authors refer to "the resting potential in I2". Do they mean V(I2) in resting state? If yes, Figure 3 does not show V(I2) in any panel. Fig3 C and F show V(I1). In the sentence "The increase in Kir channels conductance hyperpolarized the resting potential in I2, since in this region bistability was not observed I1 = I2" does the 'since' indicate causality? I.e. the effect of Kir would have been different, if this region was bistable? If so, why? Should there

be brackets around I1 = I2? The authors say “In this scenario, I1 was lower than I2” but isn’t that true by definition?

Furthermore, I cannot follow the authors' reasoning for why KM, with its positive differential conductance, would reduce the bistability window size. Consider the following thought experiment:

We start with a cell and inject current I_2 while the cell is in resting state. We then slowly add KM channels. The KM channels contribute a hyperpolarizing current, which we cancel by increasing the injected current. The new current I_2' is larger than I_2.

Now we return to the original cell and repeat the procedure at I_1. As KM is added, we again increase the injected current to maintain the resting state. The new current is I_1'.

If the same KM conductance is added in both scenarios, by how much must we increase the current in each case? Because $V(I_1) < V(I_2)$, and since KM has a positive slope conductance, KM is more conductive at I2 than at I1. Additionally the distance to the potassium reversal potential is higher at $V(I_2)$ than $V(I_1)$. Both the positive slope and the more depolarized membrane potential boost I_2’ more than they boost I_1. Therefore:

$I_2' - I_2 > I_1' - I_1$

which implies:

$I_2' - I_1' > I_2 - I_1$

In other words, from this steady-state perspective, the bistability window should expand, not shrink. Could the authors clarify this? The authors refer to equation (1) in their explanation. Rather than using prose to derive conclusions from this equation, it may be easier if their conclusions were derived mathematically.

Major 3: presentation of the bifurcation analysis.

I appreciate the added brief explanations, but in all honesty, I do not think the addition provides any clarity. Can the authors annotate each respective bifurcation type and collision in the figure? Could they work on providing an intuitive understanding of what the presence of this type of bifurcation means for the behavior of the neuron and how they arrive at this conclusion?

Major 4: effect of plateau size on afterdischarges and robustness to noise is insuficciently explored. The authors find that in their CaL+Kir channel model, the model is able to transition between resting and spiking states, whereas in the CaL+KM model, the model essentially never transitions back to resting. This is a very interesting analysis, but quantification of this phenomenon lacks behind compared to the quantification of the bistability window size. The authors say “Therefore, we consider $\bar{g}$KM-bistability more fragile than $\bar{g}$Kir-bistability.”, but this claim needs to be corroborated further. Are there other variables that can modify the weight of robustness/fragility of the resting vs. spiking state? One parameter that comes to mind is the

base-current on top of which noise is added. Currently, the base current is chosen to be (I1+I2)/2. But would moving the base current towards I1 or I2 have an effect on state robustness? The authors should provide further analysis about the relationship between plateau potentials, the occurrence of afterdischarges, and the robustness of spiking vs. resting states. Line 680f: "The strong bistability created following the simultaneous increase in both CaL and Kir channels conductances resulted in the generation of plateau potentials and long-lasting afterdischarges robust to perturbations in input current and intrinsic variability." The authors show several examples of long afterdischarges. The longest afterdischarge they show is in Fig. 2A – a model with KM + CaL (not Kir). The tendency of the KM model to get stuck in the spiking state is also apparent in Fig. 6. Thus, it appears one could also arrive at the opposite conclusion – that plateau potentials may actually shorten afterdischarges. To investigate this, the authors could perform a bifurcation analysis for all models shown in Fig. 4 and for each model measure the existence and size of the plateau, the duration of afterdischarges, and the state switching probabilities under noisy input currents. Such a dataset could help idenitfy if the authors interpretation "Thus, plateau potentials might be needed to enhance the resting state robustness to perturbations " (line 690) is correct. It it is correct, one would predict that the larger the plateau size of each respective model, the higher the probability for a spiking-> resting transition.

Minor 1: Terminology

I1 and I2 are referred to inconsistently. For example:

- threshold current for spiking. Is this I_1?
- the resting potential in I_2. Is this $V(I_2)$?
- maximum resting potential (line 297). Is this $V(I_2)$?

I suggest the authors define the currents once (e.g. I1 as current above which spiking appears, and I2 as current above which resting disappears) and from then on use symbols to refer to them.

Furthermore, I find the authors use of the term "resting potential" extremely confusing. Typically, the resting potential refers to the membrane potential in the absence of stimulation. If possible, I suggest that the authors avoid this term and introduce symbols (e.g. V(I1) and V(I2)?).

Minor 2: Figures

- 3 and 5: In the heatmaps, the authors should annotate (e.g. by encircling the respective set of models)
  - which models meet their criteria for physiological plausibility
    - which models have a V(I1) below E_K.
  - which models do not show bistability
- 6C,F: Do the plots show all data? I would expect that each line extends to the full range (0 to 6).
- 8: Please add a scale to the y axes.
- 10B: The line in the lower panel is incomplete (stops at ~5sec)
- 10E,G,F,H and 11:
  - The curved dashed line should be redrawn. I have raised this issue before, but it still persists. The dashes have different gaps, and for the most part merge into a solid line. If this is intentional then the authors need to provide a legend that indicates the meaning of a black solid line. I assume that it is not intentional, as I noticed that the dashed lines have a more clear seperation in the appended full size figure compared to the figure that is embedded in text. This may hint at an issue with how the figure is exported.
  - All plots show a dashed line around -30mV. Is this indeed a saddle node?
  - I want to suggest to indicate on each panel which currents are present (similar to Fig 3 which clearly labels the figures as CaL+KM and CaL+Kir).
  - The main text mentions various types of bifurcations / collisions. These should be annotated in the figure.

Minor 3: Writing

Overall, I find the manuscript difficult to read. I suggest the authors revise for conciseness and clarity, and the removal of redundancy. AI writing tools (e.g. Claude, ChatGPT) may be helpful for improving flow and identifying ambiguous wording.
