# Supplementary material for: Inward rectifier potassium channels interact with calcium channels to promote robust and physiological bistability
Source: PLoS Comput Biol. 2026 Jul 28;22(7):e1014549. doi: 10.1371/journal.pcbi.1014549 (PMC13441080; doi:10.1371/journal.pcbi.1014549)
Supplement: Supplementary file 12 [file pcbi.1014549.s009.pdf]

Reply to the Reviewers' Comments for Submission PCOMPBIOL-D-25-01035, Kir and Ca channels promote robust bistability

Anaëlle De Worm, Guillaume Drion, Pierre Sacré

Comments by Editor

E.1 *Please make sure to address the major concerns by the reviewers regarding a better exploration of the model and its parameters to get a better mechanistic understanding of what causes bistability. Also, the biological plausibility must be shown convincingly in a revised version of the manuscript.*

We thank the Editor and Reviewers for their feedback and constructive suggestions. Please find below our detailed responses and corresponding modifications in the manuscript. Main changes in the paper structure are:

- Validation of our results in a published two-compartment conductance-based model of deep projection neurons [5] that was parametrized based on experimental data and matches the published ranges of firing frequency of deep projection neurons [6] (new Fig. 5).
- Addition of an analysis of the combined effect of KM and Kir on calcium channel-induced bistability (new Fig. 4).
- Increase of the parameter space considered in our study, including in the absence of calcium channels (see Supplementary information).
- Main revision of the Discussion section.
- Added quantifications of effects and comparison with available experimental data.
- Homogenization of all figures, labels legends and captions. Values, units and axes were added and uniformized throughout the whole manuscript.

Comments by Reviewer 1

In the study "Inward rectifier potassium channels interact with calcium channels to promote robust and physiological bistability," the authors investigate a potentially important mechanism underlying the sensitization of nociceptive projection neurons in the spinal cord following intense pain stimuli. Using single-compartment models, they explore how L-type calcium channels and potassium channels (Kir vs. KM) may contribute to bistability—a phenomenon that could support persistent neuronal sensitization. While the topic is compelling and relevant, the study in its current form has several major limitations.

Major issues

R1.1 *Lack of Comparison to Empirical Data.*

The authors do not compare their model results to electrophysiological data from the neuron type they investigate. This raises concerns about the physiological relevance of their findings. For instance, the firing rates reported in Figure 1C reach 100–200 Hz, whereas Monteiro et al. — cited in the manuscript — report typical firing rates of 10–20 Hz in rat dorsal horn neurons. This discrepancy calls into question whether the model reflects realistic neuronal dynamics.

Furthermore, the authors frequently distinguish between "physiological" and "nonphysiological" bistability, but this distinction is not clearly defined or grounded in empirical observations. The

study uses the "bistability window" — the range of input currents for which the neuron's output depends on its initial condition — as a measure to quantify bistability. However, the manuscript does not clarify what range of bistability windows would be considered physiological.

To strengthen the study, the authors should define their use of the term "physiological", provide quantifications of their models responses (e.g., firing rates, bistability windows, AP amplitudes, AP width, amplitudes of the plateau potential), and compare them to empirical data from dorsal horn neurons.

We better defined *physiological bistability* (and *unphysiological bistability*), which refers to the fact that the resting potentials at which bistability can be observed lie within physiological ranges of values (see Introduction). We explained that the resting potentials are considered physiological if they are above the reversal potential of potassium, which matches experimental data [1-4].

The physiological range of bistability window sizes is more difficult to quantify based on the published experimental data of deep projection neurons, due to a lack of quantitative experimental studies on the subject. Nevertheless, we discussed this question and proposed some hypotheses drawn from the deep DHN recordings showing sustained afterdischarges (see Discussion).

In addition, following reviewers' comments and suggestions, we validated the phenomenological results obtained on the minimal model (i.e. containing the minimal set of conductances required to exhibit the analyzed properties) in a published two-compartment conductance-based model of deep projection neurons [5] that was parametrized based on experimental data and matches the published ranges of firing frequency of deep projection neurons [6].The results of this analysis strengthen the physiological relevance of our phenomenological findings, as they extended to this more complex and relevant model (see new Fig. 5 and new associated Section).

R1.2 *Limited Exploration of Parameter Space*

Throughout most of the study, the authors use a fixed base model to which either CaL + Kir or CaL + KM channels are added. However, it remains unclear to what extent the results depend on the specific expression levels of the other ion channels included in the model, and whether the findings would fundamentally change in the presence of additional conductances that are expressed in dorsal horn neurons. While the use of a minimal model to explore how bistability can arise in principle is understandable (and I consider in particular their bifurcation analysis as a strength of their study), the authors should also construct a model that reproduces the characteristic electrophysiological properties of rat dorsal horn neurons and verify that their main conclusions generalize to this more realistic scenario.

We extended the bistability analysis in a two-compartment conductance-based model previously published which was fitted on experimental data of rat deep projection neurons [5]. This model includes Na, K, fast CaL, slow CaL, Kir, KCa and CAN channels, representing a more realistic scenario. The findings generalized to this more complex model. Additionally, we added a discussion about the possible effect of the additional ion channels considered (see new Fig. 5 and new associated Section).

In addition, as also suggested by Reviewer 2, we extended our analysis in the minimal model in the presence of both Kir and KM (see new Fig. 4).

Finally, we extended the explored parameter space (see Supplementary information, S1 Fig. 1, S2 Fig. 1, 2, S3 Fig. 1, S4 Fig. 1, S5 Fig. 1).

R1.3 *Inconsistent and Ambiguous Terminology Terminology varies throughout the manuscript, and several key terms are not clearly defined. This inconsistency creates confusion and hinders interpretation of the results.*

For example, the following phrases appear in diofferent sections of the manuscript and seem to describe the same concept:

- *"trade-off between bistability and the plausibility of resting states" (Abstract)*
- *"trade-off between bistability and the plausibility of resting potentials" (Author summary)*
- *"trade-off between bistability robustness and resting potential adjustment" (line 27)*
- *"trade-off between the bistability window size and the plausibility of the resting potentials" (line 141)*

- *"trade-off exists between the bistability window size and the resting potential adjustment" (line 146)*
- *"trade-off observed between the bistability window size and the adjustment of its equilibrium potentials" (line 355)*

Do all of these refer to the same? The manuscript should consistently use a single term or clearly explain the distinctions. Additionally, does the use of "resting potential" and "equilibrium potential" imply that the authors treat them interchangeably? If so, this deviates from standard definitions — typically, resting potential refers to the steady-state membrane potential in the absence of stimulation , while equilibrium potential refers to the Nernst potential of a particular ion species. Fig 3 reports resting potentials far below the reversal potential of potassium channels, suggesting that the authors definition of these terms indeed deviates from the commonly used definition. I assume the authors refer to the voltage observed at current I_1?

Similar concerns apply to other vague or inconsistently used terms, including:

- *"high level of bistability" (line 106)*
- *"weakened bistability" / "strengthened bistability" (line 52)*
- *"robust bistability"*
- *"reinforce bistability" (line 56)*
- *"classical potassium channels"*

The authors should rewrite the manuscript to ensure each term is clearly defined before it is used for the first time and aligns with the definitions commonly used in the field, and that terminology is kept constant throughout the manuscript.

We thank the reviewer for this comment. We removed the variability in terminology that the reviewer pointed out, and adapted the manuscript to make it more consistent and unambiguous. In the first version of the manuscript, we unintentionally deviated from the standard definitions commonly used in the field, as the reviewer mentioned. These ambiguities were corrected as well throughout the manuscript.

Minor issues

R1.4 *Missing Units on Figure Axes: Several figure panels don't have any units on their axis (e.g. Fig 3)*

Values, units and axes were added and uniformized throughout the whole manuscript.

R1.5 *Several legends do not match the content of the figure. For instance, in Figure 8E, the legend refers to a "saddle node" line style that is not clearly visible in the plot. The line styles for "stable node" and "cycle extrema" appear nearly identical — possibly due to minimal dfferences in color shade? — making them hard to distinguish. Similar issues are present in other panels of Figure 8, as well as in Figure 9C–D. The authors should ensure that all line types, colors, and markers are distinct and clearly match the legend.*

We have checked and revised figures, legends and captions to ensure their readability.

R1.6 *Use of Jargon. The manuscript uses technical terms from bifurcation theory (e.g., "saddle-node on invariant circle bifurcation," "saddle-homoclinic bifurcation") without explanation. While appropriate for readers with a mathematical modeling background, such terminology may limit accessibility for neuroscientists with primarily experimental or biological expertise.*

We added a short definition of these two types of bifurcation in the Results section when they are introduced and added related references.

Comments by Reviewer 2

In this paper, the authors investigated the interaction between voltage-gated calcium and potassium channels and their promotion of robust and physiological bistability in a conductance-based model of a dorsal horn neuron. Bistability is a problem that has been investigated in several different studies. However, the underlying mechanisms are still not fully understood. Therefore, this paper raises a valid issue, which the authors analyse at different levels. The paper is clearly written; however, I have some suggestions for improvement and some questions for the authors to clarify. Overall, I think the paper is suited for PLOS CB.

Major issues

R2.1 *The authors analysed and compared combinations of the CaL and KM channels, as well as the CaL and Kir channels. What about the combination of CaL + KM + Kir? Could you explain why you only tested CaL + KM and CaL + Kir, given that KM and Kir can be co-expressed in the same cell? Alternatively, could you include simulations for the CaL + KM + Kir combination in a single model?*

As suggested by the reviewer, we studied the effects of combining CaL, KM and Kir channels in the model, and analyzed the bistability window size and the resting potentials in the bistability window. The results of this analysis were added to the end of the first section of the Results (see new Fig. 4). Considering both KM and Kir together adds a degree of freedom, which permits to tune bistability window size and resting potential independently.

In addition, we validated our results in a published two-compartment conductance-based model of deep projection neurons [5], as also suggested by Reviewer 1 (see new Fig. 5 and associated Section).

R2.2 *Lines 223–224: The authors write that spiking and resting in gKirk bistability were more robust than resting in gKM bistability. While I agree that gKirk bistability at rest is more robust than gKM bistability, gKM spiking was not corrupted by increased noise as it was in the case of gKM. Could you please clarify this?*

The reviewer is right with respect to the robustness of $\bar{g}_{\mathrm{KM}}$ spiking. We clarified this question of contrasting the robustness of $\bar{g}_{\mathrm{KM}}$-bistability and $\bar{g}_{\mathrm{Kir}}$-bistability by highlighting the fact that the resting state is almost unreachable in $\bar{g}_{\mathrm{KM}}$-bistability.

R2.3 *Lines 367–371: The authors describe the effect of pCaL and gKir on the plateau. However, in the case of gKM, they mention an increase in resting potential, and a plateau is also visible in this case. Could you clarify this, as well as the effect that pCaL and gKir have on the resting potential? How is the plateau measured (quantified)? Is it based on duration?*

We defined plateau potentials as a clear separation between the range of potentials encompassed by spiking and the resting potentials below I_1. We have clarified our definition in the corresponding section. We further discussed the origin of this difference in plateau potentials using the shape of the differential conductances of KM and Kir (see new Fig. 8 and associated text).

R2.4 *Line 414: The authors state that plateau potentials were absent when CaL channels were combined with KM channels. However, figure 9B shows a short plateau. Could you please clarify this?*

The manuscript was indeed ambiguous in this regard. We have clarified the differences between plateau potentials and afterdischarges. Plateau potentials were defined as above, whereas afterdischarge concerns the continuation of spiking after the end of the depolarization pulse.

R2.5 *Discussion: The authors should compare their results with those of other papers more. The current version is more of a summary of the results, as they only reference other papers in three places (lines 437, 461 and 486). Of these, they report twice that it is in line with a different paper (lines 437 and 461).*

We included a paragraph that focused on the results obtained by repeating our analysis of bistability with a published two-compartment model of deep projection neurons, closer to experimental data due to its complexity, as suggested by another reviewer. This analysis showed that our findings generalize to this more complex model. The possible impact of the additional ion channels

considered in this model were discussed based on several studies considering sustained afterdischarges. In addition, we included a note on several neurotransmitters in the dorsal horn that were recently shown to modulate Kir channels. Finally, we strongly revised and extended the discussion to better connect our results with previous experimental and computational work.

R2.6 *Supporting materials: The supporting materials are not referenced in the main text. The idea of testing different models of* Ca^{2+} *channels is valid. However, the author should also mention this in the main text (perhaps in the discussion section).*

We thank the reviewer for this comment. Each appendix was referenced in the manuscript where appropriated.

Minor issues

R2.7 *Figures in general: The authors should agree on how to display graphs of voltage traces and injected currents. It is better to label the full y-axis as in Fig. 2A, and the x-axis similarly. In addition, is it necessary to plot the full simulation? A shorter version showing the most important parts would be more informative.*

We have checked and revised figures, legends and captions to ensure their readability. We have also added and uniformized values, units, axes and simulation times throughout the whole manuscript.

R2.8 *Fig. 1: If this is a single simulation, it would be appropriate to include specific values for I1 and I2 in the graphs and text to help with understanding.*

We have added specific values through the entire manuscript.

R2.9 *It might be appropriate to mark the simulation in Figures 1A and 1B as points in Figure 1C, so that the differences are visually apparent and it is clear at which currents bistability occurs.*

We have followed the reviewer's suggestion by adding markers in the fI curves, which indeed improves the readibility of the figure.

R2.10 *Do the colours in Figures 1A and 1B represent the same amplitudes of the injected currents? It is not clear from the text.*

We clarified this in the figure by adding explicit values, as well as in the text and in the caption.

R2.11 *From the Methods section, I understand that you ran four simulations for Figure 1. How do you calculate the curve in Fig. 1C? In my opinion, four simulations/points are not sufficient for this purpose. As mentioned above, I would prefer to mark the values from the simulations.*

We indeed ran 4 simulations for Fig. 1 A-B to show bistability. As you suggested, we added markers corresponding to the currents used in Fig. 1A-B to Fig. 1C, which correspond to the final state observed (either resting or spiking). The fI curve in Fig. 1C(top) was computed by simulating 129 data points between -2.65 and $1\,\mu A/cm^2$ in the case of Fig.1C. We have clarified this in the fourth subsection of the Methods, and referenced to this subsection in the text.

R2.12 *In lines 106–107, you state that calcium channels provide a high level of bistability. What do you mean by 'high level'? Could you clarify or quantify this?*

We appreciate this comment, which was in line with comments from other reviewers. We have clarified qualitative descriptions by using quantifiable measures throughout the whole manuscript.

R2.13 *Why did you apply a different duration of input current in Fig. 1 (almost the whole simulation) than in Fig. 2 (only 100 ms)? Are these two scenarios comparable? Please, clarify.*

We indeed did not appropriately explain the equivalence between these scenarios in the first version of the manuscript. We clarified this by comparing these two scenarios in the main text.

R2.14 *Fig. 2A, B: The y-axis is fully labelled, but the x-axis is not. Please harmonise it. Again, I don't think it is necessary to show the full 120 s simulation. The lowest voltage is around -80 mV. There is no need to have a tick at -150 mV (same for Fig. 2C and 2F).*

We made the appropriate adjustments to the figure.

R2.15 *For the bistability windows, I would create a summary table showing I1, I2 and delta I for each scenario. This will help to understand how it changes with different modifications.*

We appreciate your perspective and we agree with the lack of clear quantifications of I_1, I_2 and ΔI obtained with specific sets of maximal conductances in the Results of first version of the manuscript. Based on your and other Reviewers' comments, we now display I_1, I_2, and ΔI and their numerical values in the figures showing bistability windows.

R2.16 *How many simulations did you run for the curves in Figures 2B, 2C, 2E and 2F? How did you calculate these curves? Please, clarify. Again, mark the simulated values.*

We clarified these computations in the Methods and added a reference to the corresponding Methods subsection. We also added the number of data points used to compute the curves shown in each figure. We agree that the previous version of Fig. 2 did not allow to relate the current value used in Fig2A(resp. D) with the curves displayed in Fig.2B-C(resp. E-F). In order to standardize the template for the voltage traces (as suggested in R2.7), we displayed the absolute value of current during the pulse.

R2.17 *Lines 151–152: Could you quantify or support your quotation in some way, because in my opinion the effect of KM on the bistability window is minimal?*

We added the corresponding quantification in the main text.

R2.18 *Fig. 3: What step did you use to change the conductance? For example, you started with gKM=0 and increased it in steps to gKM=0.3. It is useful information for evaluating the sampling and the number of simulations you run. In addition, it would be useful to see how I1 changed for the different pCa+gKM and pCa+gKir combinations.*

We added the step used for the ranges of $\bar{g}_{\mathrm{KM}}$, $\bar{g}_{\mathrm{Kir}}$, and $\bar{p}_{\mathrm{CaL}}$ to create the heatmaps shown in Fig. 3. In addition, we added the heatmap of I_1.

R2.19 *Fig. 6: More ticks are needed on the y-axis and x-axis in all subplots. I would either add ticks for 0 mV or use denser ticking (for example, 20 mV).*

We have revised Fig. 6 (new Fig. 8) accordingly.

R2.20 *Fig. 7: I would add the exact values of I1 and I2 to all subplots for easier understanding.*

We have revised Fig. 7 (new Fig. 9) accordingly.

R2.21 *Fig. 8: Use the exact values of I1. Add more ticks to the y-axis (A–D). Plotting only the most important part of the voltage traces would be more informative than plotting the full simulation as is currently done. Does the dashed line represent I1? If so, this should be noted in the figure caption.*

We have revised Fig. 8 (new Fig. 10) accordingly.

R2.22 *Line 365: Could you clarify what you mean by 'after a significant increase in pCaL'?*

We added the numerical value in the main text to clarify this statement.

Comments by Reviewer 3

The article illustrates the effect of L-type calcium channels in combination with different potassium channels on membrane voltage bistability in a computational model neuron. It agues for a trade off between membrane bistability and physiological resting states. In particular, the authors report that Kir and KM potassium channels have disparate effects on bistability, while stating the exact mechanisms remain unclear.

R3.1 *The article, does not provide any mechanistic insight into the observed phenomena. It is also not clear how idiosyncratic the observed effects are to the specific model in use. The article does not provide a rigorous sensitivity analysis of the results, nor analytical arguments. Consequently we would suggest the manuscript is more suited for PLOS ONE.*

We appreciate the Reviewer's perspective, and we attempted to improve our paper in these regards in the revised version.

The submitted manuscript indeed focused on a minimal model, and suffered from a lack of generalization of our findings to other, already published models. This concern was raised by the other reviewers as well. We have added an analysis of the results in a published two-compartment conductance-based model of deep projection neurons [5], and found that our findings were consistent across models (see new Fig. 5 and associated new Section). We also extended the sensitivity analysis by extending the range of the considered parameter space beyond our previous focus (see Supplementary information).

Our goal with the minimal model was to allow for tractable analytical and mechanistic analyses, such as the differential conductance analysis of new Fig. 8, 9 and bifurcation analyses of new Fig. 10, 11. We extended the mechanistic interpretation of those results in the text associated to Fig. 8. The new connection between these results and those of the published two-compartment conductance-based model of deep projection neurons suggests that the described mechanisms are of interest for deep projection neuron electrophysiology.

R3.2 *The main conclusion of Fig 1. is that the addition of L-type calcium channels introduces bistability into a neuron model. I would be cleaner if the results, particularly the f-I curve and the resting voltages in panel C would also contain the date from the model without L-type calcium channel for comparison. After all, the model could already show bistability to begin with.*

We indeed did not analyze the bistability without calcium channels in the previous version. We added the fI curve and the VI curve of the model without calcium channels in S1 Fig. 1, and referred to these data in the main by mentioning that the model did not show bistability before the addition of CaL channels.

R3.3 *Could the authors provide a brief description of the mechanism behind the strong resting voltage reduction of the Calcium channels.*

We added a mechanistic interpretation behind the strong resting voltage reduction of the calcium channels in the main text related to Fig. 8. This interpretation uses the concepts derived in Fig. 8, which justifies its position in the manuscript.

R3.4 *From the writing in this first results section it is not clear how idiosyncratic to the specific model the effects are. Furthermore, other manipulations of ion channel conductances can also induce bistability. For example altering only the conductances if I_(Na) and I_(KDR) would for some values also lead to bistability.*

To mitigate the possibility that the mechanisms and results are specific to the minimal model used in this work, we have tested and validated the results using a published two-compartment conductance-based model of deep projection neurons [5]. We also focused on calcium channel-dependent bistability, as opposed to the bistability linked to a Hopf bifurcation in a Na-K model. The calcium channel-dependent bistability has indeed been shown to be prominent in deep projection neurons in both experimental and modeling works. Our work focused more on the added effects of Kir and KM. In addition, we showed that the minimal model did not exhibit bistability in the absence of calcium channels (see S1 Fig. 1), which is also the case for the two-compartment model.

R3.5 *Could the authors supply citations for the statement that "in numerous, instances, the increase in the conductance of calcium channels is assumed to be coupled with an increase ... of ... potassium channels"?*

The relevant citations have been added in the main text.

R3.6 *All the x-axis of Fig. 1C and Fig. 2B, E are different in scaling and thus range Δf are hard to compare.*

We have checked and revised figures, legends and captions to ensure their readability. We have also added and standardized values, units, axes and simulation times throughout the whole manuscript.

R3.7 *On the one hand, the authors mention that in the presence of M-type potassium channels the model fails to maintain spiking and bistability can not be maintained, on the other hand panel B shows a bistable f-I curve. In the text it is stated that range "appear narrower" after the addition of M-type potassium channels. How is this then defined and can this please be quantified.*

We have clarified this effect and provided quantifications throughout the whole manusript. In addition, we have studied the effect of the combination of Kir and KM and calcium channel-dependent bistability (see new Fig. 4).

R3.8 *What is the mechanism of the spike failure? M-type potassium currents typically lead to spike frequency adaptation and can terminate spiking. They may however be still bistable in the un-adapted state.*

We have indeed studied the bistable properties in the model including all adaptation mechanisms. Spike failure mechanisms include the slow activation of M-type potassium current, but also the ultraslow inactivation of calcium channels. We have added this mechanistic interpretation in the main text associated to new Fig. 2A.

In addition, we have shown in new Fig. 8 that M-type potassium currents add a positive differential conductance to the model, which counterbalance the negative differential conductance brought by calcium channels, hence reducing the bistable region.

R3.9 *For the reader to appreciate the different properties it would be useful to plot the steady state I-V relations and dynamic currents during the AP of Kir vs M-type as well as their time constants.*

Following Reviewer's advice, we have added the dynamic currents of CaL, Kir and KM in Fig. 2A/D. The steady-state IV curves are provided in Fig. 8.

R3.10 *Fig. 3 seems to show that the depolarising CaL channel hyperpolarises the resting potential, while the repolarising potassium channels depolarise the resting potential. Is this due to the model relying on the Goldman-Hodgekin-Katz formalism?*

CaL being a depolarizing current, it makes the neuron excitable/spiking at lower potentials. As a results, resting states can only be reached for more hyperpolarized values. Conversely, potassium channels make the neuron less excitable at lower potentials, which requires to raise the membrane potential to reach excitability and spiking. This mechanism is independent from the formalism chosen to describe current dynamics. We have clarified this mechanism in the main text related to new Fig. 8.

References

1. *Morisset V, Nagy F. Ionic basis for plateau potentials in deep dorsal horn neurons of the rat spinal cord. Journal of Neuroscience. 1999 Sep 1;19(17):7309–16. Available from: https://doi.org/10.1523/jneurosci.19-17-07309.1999*

2. *Derjean D, Bertrand S, Masson GL, Landry M, Morisset V, Nagy F. Dynamic balance of metabotropic inputs causes dorsal horn neurons to switch functional states. Nature Neuroscience. 2003 Feb 18;6(3):274–81. Available from: https://doi.org/10.1038/nn1016*

3. *Reali C, Russo RE. An integrated spinal cord–hindlimbs preparation for studying the role of intrinsic properties in somatosensory information processing. Journal of Neuroscience Methods. 2004 Nov 12;142(2):317–26. Available from: https://doi.org/10.1016/j.jneumeth.2004.09.006*

4. *Reali C, Fossat P, Landry M, Russo RE, Nagy F. Intrinsic membrane properties of spinal dorsal horn neurones modulate nociceptive information processing in vivo. The Journal of Physiology. 2011 Apr 12;589(11):2733–43. Available from: https://doi.org/10.1113/jphysiol.2011.207712*

5. *Franc YL, Masson GL. Multiple firing patterns in deep dorsal horn neurons of the spinal cord: Computational analysis of mechanisms and functional implications. Journal of Neurophysiology. 2010 Jul 29;104(4):1978–96. Available from: https://doi.org/10.1152/jn.00919.2009*

6. *Zhang TC, Janik JJ, Grill WM. Modeling effects of spinal cord stimulation on wide-dynamic range dorsal horn neurons: influence of stimulation frequency and GABAergic inhibition. Journal of Neurophysiology. 2014 May 1;112(3):552–67. Available from: https://doi.org/10.1152/jn.00254.2014*
