# Supplementary material for: Inward rectifier potassium channels interact with calcium channels to promote robust and physiological bistability
Source: PLoS Comput Biol. 2026 Jul 28;22(7):e1014549. doi: 10.1371/journal.pcbi.1014549 (PMC13441080; doi:10.1371/journal.pcbi.1014549)
Supplement: Supplementary file 14 [file pcbi.1014549.s011.pdf]

Reply to the Reviewers' Comments for Submission PCOMPBIOL-D-25-01035R1, Kir and Ca channels promote robust bistability

Anaëlle De Worm, Guillaume Drion, Pierre Sacré

Comments by Editor

E.1 *Sorry for the delay. There have been major technical problems with the editorial manager. They are still unsolved. Please address the important concerns by Reviewer 1. If you do not see the attached pdf (I don't) please let the PLoS staff know.*

We thank the Reviewers for their second-round assessments. Below we reproduce each comment in full (in italics) and detail our response (in blue). Line and figure numbers refer to the revised manuscript unless otherwise stated.

Comments by Reviewer 2

R2.1 *I have carefully read the revised manuscript. The authors have adequately addressed all of my previous questions and have satisfactorily clarified the issues I raised. The figures are now clearer and more readable, the overall text has improved, and the discussion has been strengthened. I have no further comments and recommend the manuscript for acceptance.*

We thank the Reviewer for the careful reading of both versions of the manuscript and for the recommendation for acceptance.

Comments by Reviewer 1

R1.0 *The authors have significantly extended and improved the manuscript. Some of my previous concerns have been fully addressed, others remain, and a few new issues have emerged. With additional analysis, reframing to better match the data, and a concerted effort to improve clarity and conciseness, I believe the manuscript would be ready for acceptance.*

We are pleased that the previous round of revisions has addressed several of the Reviewer's concerns, and that the remaining points are largely about framing and presentation. In this revision we have addressed each of the four major points and implemented every minor request. The manuscript has also been rewritten for conciseness, as detailed in the response to Minor 3.

Major issues

R1.1 *The authors binary presentation of "Kir promotes bistability, KM impairs bistability" is not supported by the data.*

In Figure 3, the authors generate models with different parameter values for the maximal conductance of CaL + KM vs CaL + Kir channels and then quantify the bistability window size for each model. The figure shows that the bistability window size is largely insensitive to potassium channel expression and is dominated by CaL expression. KM has almost no effect (for high CaL values, it slightly reduces the window size, for low CaL values it may slightly increase it – but this hard to tell from the colormap, I suggest the authors include isopotential lines). Panel 3D, shows that Kir

does have a stronger effect, which clearly matches what the authors say (the more Kir the larger the bistability window), but the effect of Kir still remains far below the effect of CaL.

The authors should highlight the parameter regions in in which their criteria for robust bistability and physiological membrane potentials are met. If I search e.g. for models with a window size > 2 and a V(I1) > -70mV, it seems that there are many solutions for both the CaL+Kir and the CaL+KM channel.

This also calls into question the usefulness of the concept of a "trade-off between bistability and the plausibility of resting states", as the authors clearly show that bistability with plausible membrane potentials can emerge with either channel. If the goal is simply to achieve bistability wihtin physiological constraints (not to maximize window size), then the trade-off dissolves — both channels can get you there, with window sizes that are about similar. The trade-off only matters if maximizing window size is biologically meaningful, which the authors haven't established.

The binary narrative of contrasting Kir with KM seems particularly extreme in the figure legend of Figure 2, Panel A, where the authors say "When adding KM [. . .] the neuron response to a pulse of similar pulse amplitude as in Fig 1A shows that bistability [. . .] is not observed anymore." And for Panel D: "Replacing KM channels with Kir channels restores bistability between resting and spiking in the neuron response to a pulse of similar amplitude as A". This is misleading. The authors chose to give a current stimulus that jumps from 0.9 to 3.9 and back for Panel A and a stimulus that jumps form -0.6 to 2.5 and back for Panel D. Importantly, 0.9 in Panel A is clearly below the bistability window for the KM model (I1 is 1.2 according to Panel 2B). In contrast, -0.6, chosen for the Kir model, is right at the border within its bistability window. It is thus not surprising that the model returns to a stable resting state in Panel A. Had the authors replaced the stimulus protocols such that the base current for the Kir example is below I1 and the base current for the KM example is between I1 and I2, the figure would likely have demonstrate the opposite result.

Essentially, I would suggest to drop the binary framing, and instead focus on how the potassium channel kinetics can modulate bistability. The interpretation of modulation seems also adequate given Fig. 5, which shows the effect of Kir and CaL added to a more realistic model. Again, CaL shows up as the major determinant of bistability (with the bistability window size jumping up as soon as CaL reaches a threshold) whereas Kir conductance plays a minor role. I suggest to highlight models that meet the authors criteria also in this figure.

While I appreciate the effort of testing their prediction of the effect of Kir channels in a more realistic model, the authors did not perform a similar analysis combining KM with CaL in the more realistic model. Given that the simplified model showed viable parameter combinations for both Kir and KM, it seems likely that solutions meeting the authors' criteria would also exist for KM in the full model.

The Reviewer raises three points that we take in turn: the framing of Kir vs. KM, the case of Figure 2, and the absence of a KM analysis in the two-compartment model. We have refined the framing and acted on each point, while preserving the substantive claim that CaL+Kir is functionally favored over CaL+KM, a claim that we believe the data clearly support, but which we previously stated more sharply than the data warranted.

Framing. We have softened the binary framing while retaining a substantive comparison. The revised manuscript now organizes the comparison along three claims, all directly supported by the figures:

(a) CaL channels are the primary determinant of bistability; without them no bistability is observed (*S1 Appendix*).

(b) When paired with CaL, both Kir and KM channels can support bistability that is simultaneously robust and physiological.

(c) Within that joint criterion, the parameter region for robust+physiological bistability is larger for CaL+Kir than for CaL+KM, and CaL+Kir bistability is more robust to noise and to intrinsic variability. The two pairs also support qualitatively distinct excitability (plateau-generating vs. resonator-like).

The abstract, Introduction, and Discussion have been rewritten to lead with this framing.

Figures 3 and 5: isopotential lines and demarcation of the robust and physiological region. Contour lines have been added to every heatmap in Figs. 3A–F and 5G–I, as suggested.

Two red lines in Fig. 3 delimit the parameter region where bistability satisfies the joint criterion $\Delta I \geq 1.6\,\mu\text{A cm}^{-2}$ and $\overline{V}(I_1) \geq -90\,\text{mV}$. The caption states explicitly: "*The two red lines together delimit the parameter region (top right) in which bistability is simultaneously robust and physiological.*" The same demarcation is applied to Fig. 5 (red dashed line at $\Delta I = 20\,\text{pA}$, the robustness threshold estimated from published experimental data). These additions make the parameter regions explicit and visibly show the larger robust and physiological region for CaL+Kir.

Figure 2: matched stimulus protocols. We agree that the previous Fig. 2A baseline was below I_1, which prevented a direct comparison. The revised Fig. 2A,D applies the same protocol class to both models (a pulse from a baseline immediately below I_1, with pulse amplitude crossing I_1). Both panels now show a clean finite afterdischarge, before settling to a resting state. The text and legend have been rewritten accordingly: we now say "*both KM and Kir channels redress the resting potentials, and modulate bistability.*"

KM in the two-compartment model. The Reviewer is right that this analysis was missing. The new *S4 Appendix* reports a full sweep over $(\bar{p}_{\text{CaL}}, \bar{g}_{\text{KM}})$ in the two-compartment model. The results match the minimal model: KM channels slightly reduce ΔI at intermediate $\bar{p}_{\text{CaL}}$ and can suppress bistability altogether at high $\bar{p}_{\text{CaL}}$. This is referenced at the end of the two-compartment results section.

We believe the revision keeps the substance while addressing the framing concern: the paper shows that CaL+Kir is functionally favored, but no longer reads as a binary verdict.

R1.2 *The reasoning about the effect of the positive / negative differential conducantce on the bistability window size is hard to follow.*

The section is difficult to follow, in parts due to inconsistent terminology and apparent figure reference errors.

Lines 481-491: "However, because they have both a region of positive and negative slope in 482 their steady-state current, their impact on the resting potentials is not straightforward. Below -60.1 mV, the leak current is the main driver defining the resting potentials. When Kir channels are added, the peak in the steady state Kir current found at -67 mV creates a local peak in the sum of the total ion current, that is in between -60.1 and -67 mV, and closer to the latter as the Kir conductance increases. Accordingly, as shown in Fig.3C (bottom part, for p CaL 0̃), the increase in Kir channels conductance hyperpolarized the resting potential in I2, since in this region bistability was not observed I1 = I2. Respectively, when p CaL was increased to 1.725 × 10-5 cm s-1, bistability appeared, and Kir channels depolarized the resting potential in I1 (Fig.3C). In this scenario, I1 was lower than I2"

The authors refer to Figure 3C – but this panel does not show Kir channel conductance. Do the authors mean Panel 3F? The authors refer to "the resting potential in I2". Do they mean V(I2) in resting state? If yes, Figure 3 does not show V(I2) in any panel. Fig3 C and F show V(I1). In the sentence "The increase in Kir channels conductance hyperpolarized the resting potential in I2, since in this region bistability was not observed I1 = I2" does the 'since' indicate causality? I.e. the effect of Kir would have been different, if this region was bistable? If so, why? Should there be brackets around I1 = I2? The authors say "In this scenario, I1 was lower than I2" but isn't that true by definition?

Furthermore, I cannot follow the authors' reasoning for why KM, with its positive differential conductance, would reduce the bistability window size. Consider the following thought experiment:

We start with a cell and inject current I_2 while the cell is in resting state. We then slowly add KM channels. The KM channels contribute a hyperpolarizing current, which we cancel by increasing the injected current. The new current I_2' is larger than I_2.

Now we return to the original cell and repeat the procedure at I_1. As KM is added, we again increase the injected current to maintain the resting state. The new current is I_1'.

If the same KM conductance is added in both scenarios, by how much must we increase the current in each case? Because $V(I_1) < V(I_2)$, and since KM has a positive slope conductance, KM is more conductive at I2 than at I1. Additionally the distance to the potassium reversal potential is higher at $V(I_2)$ than $V(I_1)$. Both the positive slope and the more depolarized membrane potential boost I_2' more than they boost I_1. Therefore:

$I_2' - I_2 > I_1' - I_1$

which implies:

$I_2' - I_1' > I_2 - I_1$

In other words, from this steady-state perspective, the bistability window should expand, not shrink. Could the authors clarify this? The authors refer to equation (1) in their explanation. Rather than using prose to derive conclusions from this equation, it may be easier if their conclusions were derived mathematically.

The Reviewer's thought experiment correctly describes how a positive-slope potassium current would shift two resting equilibria, but it implicitly treats both I_1 and I_2 as resting states. They are not. I_1 marks the lower boundary of the bistability window, at which the *spiking limit cycle* appears (via a saddle-homoclinic bifurcation, see Fig. 11–12); I_2 marks the upper boundary, at which the upper *resting equilibrium* disappears (via a saddle-node bifurcation). Because the limit cycle and the resting equilibrium live at different membrane potentials, the comparison the Reviewer proposes cannot be carried out from the resting perspective only. This is the dynamical ingredient that was missing from the previous version of the section, and we have rewritten the section to make it the leading argument.

The rewritten version of the reasoning about the effect of positive and negative differential conductance on bistability is built around two steps:

Step 1: I_1 and I_2 are governed by different states. The revised text states (paragraph following Fig. 9): "*I_1 and I_2 are set by the appearance and disappearance of two different behaviors that are governed by distinct dynamical mechanisms.*" At I_1, the relevant state is the saddle at which the spiking limit cycle terminates; at I_2, the relevant state is the upper resting equilibrium. The effective membrane potential at which an added potassium current acts on the two behaviors is therefore different in the two cases.

Step 2: the sign of the differential conductance around the spike threshold predicts the direction of the asymmetric shift. The saddle voltage at I_1 is always *higher* than $\overline{V}(I_2)$, a fact established directly from the shared saddle-node + saddle-homoclinic structure of CaL+Kir and CaL+KM (Figs. 11F–H and 12C–D, with bifurcations annotated). It follows that:

- For a current with *positive* differential conductance around the spike threshold (KM), the outward contribution along the saddle (higher voltage) at I_1 is larger than at the lower-voltage equilibrium at I_2. I_1 therefore rises more than I_2, and the window shrinks.
- For a current with *negative* differential conductance around the spike threshold (Kir, like CaL), the outward contribution is smaller at the higher voltage. I_2 rises more than I_1, and the window enlarges.

Figure references and terminology. The reference to Fig. 3C in the Kir paragraph has been corrected to Fig. 3F. "Resting potential in I_2" has been replaced by $\overline{V}(I_2)$ throughout (and similarly $\overline{V}(I_1)$ for I_1). The clause "*since in this region bistability was not observed $I_1 = I_2$*" has been removed. The redundant sentence "*In this scenario, I_1 was lower than I_2*" has also been removed.

The section is now substantially shorter than in the previous version and revolves around a clear statement of which voltage matters at each boundary. The next sections support this statement with (i) a direct test via selective blockade of the inward/outward components of the Kir/KM current and (ii) a bifurcation argument establishing the voltage ordering. We hope this addresses both the reasoning concern and the writing concern.

R1.3 *presentation of the bifurcation analysis*

I appreciate the added brief explanations, but in all honesty, I do not think the addition provides any clarity. Can the authors annotate each respective bifurcation type and collision in the figure? Could they work on providing an intuitive understanding of what the presence of this type of bifurcation means for the behavior of the neuron and how they arrive at this conclusion?

The bifurcation section has been reworked along both axes the Reviewer suggests: figure annotation, and intuitive interpretation.

Figure annotations (Figs. 11 and 12). Each panel now carries: (i) a title indicating the channel composition ("No CaL, Kir, KM"; "CaL"; "Kir"; "KM"; "Kir + CaL"; "KM + CaL"), in the same spirit as the CaL+KM/CaL+Kir labels in Fig. 3; and (ii) direct figure labels of the

bifurcation types: "SNIC" in Fig. 11E, "SH" and "SN" in Figs. 11F–G and 12C, "Hopf" and "SN" in Figs. 11H and 12D.

Intuitive interpretation. The text now states what each bifurcation means for neuronal behavior, in plain language:

- *SNIC* (reference model, no CaL/Kir/KM): the saddle-node bifurcation occurs directly on the spiking limit cycle trajectory. Spiking appears precisely where resting disappears, so the two states never coexist, there is no bistability, and the neuron transitions directly from rest to low-frequency spiking with no voltage separation between the two states.
- *SN + SH* (CaL alone, Kir alone, CaL+Kir): low-frequency spiking is not observed anymore at a current slightly above the saddle-node because the saddle-node is not on the exact spiking limit cycle trajectory. The spiking limit cycle appears through a saddle-homoclinic bifurcation, that occurs before the saddle-node when the bistability window is non-zero, creating a current range where resting and spiking coexist. The resting branch is partially or completely separated in voltage from the spiking limit cycle, particularly at currents near the saddle-homoclimic bifurcation. Intuitively, this voltage separation means that a neuron transitioning from spiking to rest must cross a region of membrane potential that is not visited during spiking, producing a plateau potential that is observable upon hyperpolarization (Figs. 11B,C and 12A). The plateau thus reflects the robustness of the resting state: the larger the voltage separation, the harder it is for noise to drive the neuron out of rest and back into spiking. This separation is largest near I_1 and shrinks toward I_2, consistent with the current-dependent reachability and robustness observed in Figs. 7A, and Fig. 6F vs S5 Fig. 1 (see R1.4 for more details on the new additional content).
- *Hopf* (KM alone, CaL+KM): the resting equilibrium loses stability through a Hopf bifurcation, sitting inside the spiking voltage range and producing damped subthreshold oscillations on approach, consistent with resonator behavior in the sense of Izhikevich (see Chapter 7 of [1])(Fig. 11D). No plateau is thus observed upon hyperpolarization. Intuitively, this lack of voltage separation between resting and spiking states means that a neuron transitioning from spiking to resting must enter a narrow region of membrane potential encompassed by that visited during spiking, making the resting state easy to escape (Figs. 11D and 12B). This accounts for the lower robustness and reachability of the resting state in CaL+KM compared to CaL+Kir, especially on approach of the Hopf bifurcation (Fig. 6, S5 Fig. 1, and Fig. 7B) (see R1.4 for more details on the new additional content).

These interpretations are referenced where they are used: the resonator structure of CaL+KM is contrasted to the integrator structure of the SN+SH or SNIC bifurcations, which perform temporal integration of input pulses and invoked when explaining the noise-robustness asymmetry ("*This resonator behavior accounts for the reduced robustness of the resting state observed with KM channels compared to Kir channels (Figs. 6C, F and 7)*"). The saddle-homoclinic structure of CaL+Kir is also invoked thereafter to close the R1.2 argument (the saddle voltage at I_1 exceeds $\overline{V}(I_2)$).

R1.4 *effect of plateau size on afterdischarges and robustness to noise is insuficciently explored*

The authors find that in their CaL+Kir channel model, the model is able to transition between resting and spiking states, whereas in the CaL+KM model, the model essentially never transitions back to resting. This is a very interesting analysis, but quantification of this phenomenon lacks behind compared to the quantification of the bistability window size. The authors say "Therefore, we consider g KM-bistability more fragile than g Kir-bistability.", but this claim needs to be corroborated further. Are there other variables that can modify the weight of robustness/fragility of the resting vs. spiking state? One parameter that comes to mind is the base-current on top of which noise is added. Currently, the base current is chosen to be (I1+I2)/2. But would moving the base current towards I1 or I2 have an effect on state robustness? The authors should provide further analysis about the relationship between plateau potentials, the occurrence of afterdischarges, and the robustness of spiking vs. resting states. Line 680f: "The strong bistability created following the simultaneous increase in both CaL and Kir channels conductances resulted in the generation of plateau potentials and long-lasting afterdischarges robust to perturbations in input current and intrinsic variability." The authors show several examples of long afterdischarges. The longest afterdischarge they show is in Fig. 2A – a model with KM + CaL (not Kir). The tendency of the KM model to get stuck in the spiking state is also apparent in Fig. 6. Thus, it appears one could also

arrive at the opposite conclusion – that plateau potentials may actually shorten afterdischarges. To investigate this, the authors could perform a bifurcation analysis for all models shown in Fig. 4 and for each model measure the existence and size of the plateau, the duration of afterdischarges, and the state switching probabilities under noisy input currents. Such a dataset could help identify if the authors interpretation "Thus, plateau potentials might be needed to enhance the resting state robustness to perturbations " (line 690) is correct. It it is correct, one would predict that the larger the plateau size of each respective model, the higher the probability for a spiking-¿ resting transition.

The Reviewer raises two distinct questions: (1) does the base current within the window affect state robustness, and (2) what is the link between plateau potentials, afterdischarges and robustness of the spiking and resting states. To address both, we provided a new analysis and additional data, and have also corrected one overstated claim in the previous Discussion.

(a) Reachability as a current-dependent metric (new Fig. 7). The new Fig. 7 provides a noise-free, current-resolved counterpart to Fig. 6. We sweep arbitrary initial conditions $(V_0, m_{\text{ion},\infty}(V_0), h_{\text{ion},\infty}(V_0), \ldots)$ with $V_0 \in [-90, -20]\,\text{mV}$ across the entire bistability window $I \in [I_1, I_2]$ and record which state the model converges to. The maps directly show:

- In CaL+Kir bistability, resting and spiking are of comparable reachability across most of the window.
- In CaL+KM bistability, the resting basin shrinks dramatically as $I \to I_2$: near I_2, only initial conditions extremely close to $\overline{V}(I_2)$ converge to resting.
- As $I \to I_1$, the situation reverses for both pairs: resting becomes more reachable than spiking.

This directly answers the Reviewer's question about the base current: state robustness is current-dependent, and the asymmetry between resting and spiking switches direction across the window.

(b) Noise robustness at $I = I_1$ (new *S5 Appendix*). As a direct test, we repeated the noise experiment of Fig. 6 with the applied current set to I_1 instead of $(I_1 + I_2)/2$. The result confirms the reachability prediction: at $I = I_1$, resting becomes more robust than spiking for both bistability types, while resting in CaL+Kir bistability remains more robust than resting in CaL+KM bistability.

(c) Plateau, afterdischarge duration, and reachability: correcting the previous formulation. The Reviewer is right that the longest finite afterdischarge is the previous version of the manuscript was observed in Fig. 2A with CaL+KM. In the new version of the manuscript, we have matched the experimental protocols of Fig. 2A and Fig. 2D as described in R1.1, and the resulting data show a longer finite afterdischarge for CaL+Kir instead. We note that these *finite* afterdischarges reflect a transient phenomenon that can be observed below I_1, and were not the primary focus of our study, which instead examined how Kir and KM channels modulate bistability as observed through sustained afterdischarges. The previous formulation "*plateau potentials might be needed to enhance the resting state robustness to perturbations*" was therefore misleading and has been removed. We have also clarified in the manuscript that the finite afterdischarges shown in Figs. 2A and 2D occur for a baseline current below I_1.

The substantive claim that we retain (and which is supported by Figs. 6, 7 and 11–12) is conceptually different: plateau potentials are the geometric signature of a saddle-homoclinic bifurcation, and the same bifurcation structure places the resting equilibrium outside the spiking voltage range, which is what makes resting reachable and noise-robust. The plateau and the reachability are two manifestations of the same bifurcation structure; the plateau does not lengthen the afterdischarge. The Discussion now states this distinction explicitly.

(d) Full bifurcation sweep of Fig. 4 models. We considered the proposed full bifurcation sweep over the $(\bar{g}_{\text{KM}}, \bar{g}_{\text{Kir}})$ grid of Fig. 4. The four reference cases (Fig. 11: each channel alone; Fig. 12: CaL+Kir and CaL+KM) already cover the qualitative regimes that the grid spans, and intermediate $(\bar{g}_{\text{KM}}, \bar{g}_{\text{Kir}})$ combinations interpolate between these regimes continuously. Combined with the new reachability analysis of Fig. 7 and the $I = I_1$ noise analysis of *S5 Appendix*, the evidence base now spans current dependence within the window, intrinsic variability, and noise dependence at the boundary. These results are all consistent with the same bifurcation-structure interpretation. Furthermore, our results show that the plateau size in Cal+Kir directly reflects the robustness of the resting state: the larger the voltage separation, the harder it is for noise to drive the neuron out of rest and back into spiking. This separation is largest near I_1 and shrinks

toward I_2, consistent with the current-dependent reachability and robustness observed in Figs. 7A, and Fig. 6F vs S5 Fig. 1.

Minor issues

R1.5 *Terminology*

I1 and I2 are referred to inconsistently. For example:

- *threshold current for spiking. Is this I1?*
- *the resting potential in I2. Is this V(I2)?*
- *maximum resting potential (line 297). Is this V(I2)?*

I suggest the authors define the currents once (e.g. I1 as current above which spiking appears, and I2 as current above which resting disappears) and from then on use symbols to refer to them.

Furthermore, I find the authors use of the term "resting potential" extremely confusing. Typically, the resting potential refers to the membrane potential in the absence of stimulation. If possible, I suggest that the authors avoid this term and introduce symbols (e.g. V(I1) and V(I2)?).

Both suggestions have been implemented throughout the manuscript.

I_1 and I_2 are defined once in the Introduction: "*the bistability window — defined as the range of currents from I_1 (onset of spiking from rest) to I_2 (cessation of resting from spiking).*" Phrases like "threshold current for spiking" and "maximum resting potential" have been removed and replaced with I_1 and $\overline{V}(I_2)$.

The notation $\overline{V}(I)$ now denotes the steady-state membrane potential of the resting equilibrium at applied current I, and is distinguished from the conventional resting potential in the Results: "*we use $\overline{V}(I)$ to denote the steady-state membrane potential associated with the resting equilibrium at a given applied current I; this is distinct from the conventional resting potential, which refers to the membrane potential in the absence of stimulation.*" All subsequent occurrences of "the resting potential at I_1" have been replaced by $\overline{V}(I_1)$ (and similarly $\overline{V}(I_2)$, $\overline{V}(I)$).

R1.6 *Figures*

- *3 and 5: In the heatmaps, the authors should annotate (e.g. by encircling the respective set of models)*
  - *which models meet their criteria for physiological plausibility*
    - *which models have a V(I1) below E_K.*
  - *which models do not show bistability*

  As described in R1.1, white contour lines have been added to every heatmap, and two red lines in Fig. 3A–F demarcate the region where bistability is simultaneously robust ($\Delta I \geq 1.6\,\mu\mathrm{A\,cm^{-2}}$) and physiological ($\overline{V}(I_1) \geq -90\,\mathrm{mV}$, i.e. above E_K). The corresponding red dashed line in Fig. 5G marks the rescaled robustness threshold ($\Delta I = 20\,\mathrm{pA}$, estimated from published experimental data of afterdischarges; all parameter combinations in Fig. 5G–I satisfy $\overline{V}(I_1) \geq -90\,\mathrm{mV}$, so the robust+physiological region coincides with the area above the red dashed line.

- *6C,F: Do the plots show all data? I would expect that each line extends to the full range (0 to 6).*

  The simulations have been re-run and each curve now extends to the upper limit of σ. The previous truncation reflected the absence of spiking-to-resting transitions for CaL+KM rather than missing data; the figure now makes this explicit.

- *8: Please add a scale to the y axes.*

  Each panel now has explicit numeric tick labels on the y-axis, with units and parameters specified in the figure caption.

- *10B: The line in the lower panel is incomplete (stops at 5sec)*

  The display issue has been corrected and the full trace is now shown.

- *10E,G,F,H and 11:*

- *The curved dashed line should be redrawn. I have raised this issue before, but it still persists. The dashes have different gaps, and for the most part merge into a solid line. If this is intentional then the authors need to provide a legend that indicates the meaning of a black solid line. I assume that it is not intentional, as I noticed that the dashed lines have a more clear seperation in the appended full size figure compared to the figure that is embedded in text. This may hint at an issue with how the figure is exported.*
- *All plots show a dashed line around -30mV. Is this indeed a saddle node?*
- *I want to suggest to indicate on each panel which currents are present (similar to Fig 3 which clearly labels the figures as CaL+KM and CaL+Kir).*
- *The main text mentions various types of bifurcations / collisions. These should be annotated in the figure.*

– The dashed-line rendering issue was traced to PDF embedding, which caused the dashes to merge into a solid line on some PDF readers (e.g., the default macOS Preview, but not Adobe Acrobat or Microsoft Edge). The figures have been re-exported at higher resolution in a different format; the dashes are now clearly visible in both standalone and embedded forms.
– The dashed line near $-30\,\mathrm{mV}$ is the upper saddle point. The legend now labels this line as “Saddle point” alongside “Stable point” and “Cycle extrema”.
– Each panel of Figs. 11 and 12 now carries a title indicating the channel composition.
– The bifurcation types are annotated directly on the panels: “SNIC”, “SN”, “SH”, and “Hopf” as appropriate.

R1.7 *Writing*

Overall, I find the manuscript difficult to read. I suggest the authors revise for conciseness and clarity, and the removal of redundancy. AI writing tools (e.g. Claude, ChatGPT) may be helpful for improving flow and identifying ambiguous wording.

The manuscript has been rewritten throughout for conciseness and clarity, ensuring that each finding is stated once and referred back to rather than restated, removing redundant statements, and improving the flow between sections. We believe these revisions substantially improve the readability of the manuscript.

References

1. *Izhikevich, Eugene M. Dynamical Systems in Neuroscience: The Geometry of Excitability and Bursting. The MIT Press, 2006. https://doi.org/10.7551/mitpress/2526.001.0001.*
