# Supplementary material for: Inward rectifier potassium channels interact with calcium channels to promote robust and physiological bistability
Source: PLoS Comput Biol. 2026 Jul 28;22(7):e1014549. doi: 10.1371/journal.pcbi.1014549 (PMC13441080; doi:10.1371/journal.pcbi.1014549)
Supplement: Peer review history

# Peer review: Decision Letter 0

**DOI:** 10.1371/journal.pcbi.1014549.r001

**Authors:** Hermann Cuntz, Marieke Karlijn van Vugt

18 Sep 2025

PCOMPBIOL-D-25-01035

Inward rectifier potassium channels interact with calcium channels to promote robust and physiological bistability

PLOS Computational Biology

Dear Dr. De Worm,

Thank you for submitting your manuscript to PLOS Computational Biology. After careful consideration, we feel that it has merit but does not fully meet PLOS Computational Biology's publication criteria as it currently stands. Therefore, we invite you to submit a revised version of the manuscript that addresses the points raised during the review process.

Please make sure to address the major concerns by the reviewers regarding a better exploration of the model and its parameters to get a better mechanistic understanding of what causes bistability. Also, the biological plausibility must be shown convincingly in a revised version of the manuscript.

Please submit your revised manuscript within 60 days Nov 18 2025 11:59PM. If you will need more time than this to complete your revisions, please reply to this message or contact the journal office at ploscompbiol@plos.org. When you're ready to submit your revision, log on to https://www.editorialmanager.com/pcompbiol/ and select the 'Submissions Needing Revision' folder to locate your manuscript file.

Please include the following items when submitting your revised manuscript:

* A rebuttal letter that responds to each point raised by the editor and reviewer(s). You should upload this letter as a separate file labeled 'Response to Reviewers'. This file does not need to include responses to formatting updates and technical items listed in the 'Journal Requirements' section below.

* A marked-up copy of your manuscript that highlights changes made to the original version. You should upload this as a separate file labeled 'Revised Manuscript with Track Changes'.

* An unmarked version of your revised paper without tracked changes. You should upload this as a separate file labeled 'Manuscript'.

If you would like to make changes to your financial disclosure, competing interests statement, or data availability statement, please make these updates within the submission form at the time of resubmission. Guidelines for resubmitting your figure files are available below the reviewer comments at the end of this letter

We look forward to receiving your revised manuscript.

Kind regards,

Hermann Cuntz

Academic Editor

PLOS Computational Biology

Marieke van Vugt

Section Editor

PLOS Computational Biology

**Journal Requirements:**

If the reviewer comments include a recommendation to cite specific previously published works, please review and evaluate these publications to determine whether they are relevant and should be cited. There is no requirement to cite these works unless the editor has indicated otherwise.

1) Please ensure that the CRediT author contributions listed for every co-author are completed accurately and in full.

At this stage, the following Authors/Authors require contributions: Anaëlle De Worm, Guillaume Drion, and Pierre Sacré. Please ensure that the full contributions of each author are acknowledged in the "Add/Edit/Remove Authors" section of our submission form.

The list of CRediT author contributions may be found here: https://journals.plos.org/ploscompbiol/s/authorship#loc-author-contributions

2) We ask that a manuscript source file is provided at Revision. Please upload your manuscript file as a .doc, .docx, .rtf or .tex. If you are providing a .tex file, please upload it under the item type u2018LaTeX Source Fileu2019 and leave your .pdf version as the item type u2018Manuscriptu2019.

3) Please upload all main figures as separate Figure files in .tif or .eps format. For more information about how to convert and format your figure files please see our guidelines:

https://journals.plos.org/ploscompbiol/s/figures

4) Please amend your detailed Financial Disclosure statement. This is published with the article. It must therefore be completed in full sentences and contain the exact wording you wish to be published.

1) State the initials, alongside each funding source, of each author to receive each grant. For example: "This work was supported by the National Institutes of Health (####### to AM; ###### to CJ) and the National Science Foundation (###### to AM)."

2) State what role the funders took in the study. If the funders had no role in your study, please state: "The funders had no role in study design, data collection and analysis, decision to publish, or preparation of the manuscript."

3) If any authors received a salary from any of your funders, please state which authors and which funders..

If you did not receive any funding for this study, please simply state: u201cThe authors received no specific funding for this work.u201d

**Reviewers' comments:**

Reviewer's Responses to Questions

**Comments to the Authors:**

**Please note here if the review is uploaded as an attachment.**

Reviewer #1: see attachment

Reviewer #2: In this paper, the authors investigated the interaction between voltage-gated calcium and potassium channels and their promotion of robust and physiological bistability in a conductance-based model of a dorsal horn neuron. Bistability is a problem that has been investigated in several different studies. However, the underlying mechanisms are still not fully understood. Therefore, this paper raises a valid issue, which the authors analyse at different levels. The paper is clearly written; however, I have some suggestions for improvement and some questions for the authors to clarify. Overall, I think the paper is suited for PLOS CB.

Major issues:

1. The authors analysed and compared combinations of the CaL and KM channels, as well as the CaL and Kir channels. What about the combination of CaL + KM + Kir? Could you explain why you only tested CaL + KM and CaL + Kir, given that KM and Kir can be co-expressed in the same cell? Alternatively, could you include simulations for the CaL + KM + Kir combination in a single model?

2. Lines 223–224: The authors write that spiking and resting in gKirk bistability were more robust than resting in gKM bistability. While I agree that gKirk bistability at rest is more robust than gKM bistability, gKM spiking was not corrupted by increased noise as it was in the case of gKM. Could you please clarify this?

3. Lines 367–371: The authors describe the effect of pCaL and gKir on the plateau. However, in the case of gKM, they mention an increase in resting potential, and a plateau is also visible in this case. Could you clarify this, as well as the effect that pCaL and gKir have on the resting potential? How is the plateau measured (quantified)? Is it based on duration?

4. Line 414: The authors state that plateau potentials were absent when CaL channels were combined with KM channels. However, figure 9B shows a short plateau. Could you please clarify this?

5. Discussion: The authors should compare their results with those of other papers more. The current version is more of a summary of the results, as they only reference other papers in three places (lines 437, 461 and 486). Of these, they report twice that it is in line with a different paper (lines 437 and 461).

6. Supporting materials: The supporting materials are not referenced in the main text. The idea of testing different models of Ca²⁺ channels is valid. However, the author should also mention this in the main text (perhaps in the discussion section).

Minor issues

1. Figures in general: The authors should agree on how to display graphs of voltage traces and injected currents. It is better to label the full y-axis as in Fig. 2A, and the x-axis similarly. In addition, is it necessary to plot the full simulation? A shorter version showing the most important parts would be more informative.

2. Fig. 1: If this is a single simulation, it would be appropriate to include specific values for I1 and I2 in the graphs and text to help with understanding.

3. It might be appropriate to mark the simulation in Figures 1A and 1B as points in Figure 1C, so that the differences are visually apparent and it is clear at which currents bistability occurs.

4. Do the colours in Figures 1A and 1B represent the same amplitudes of the injected currents? It is not clear from the text.

5. From the Methods section, I understand that you ran four simulations for Figure 1. How do you calculate the curve in Fig. 1C? In my opinion, four simulations/points are not sufficient for this purpose. As mentioned above, I would prefer to mark the values from the simulations.

6. In lines 106–107, you state that calcium channels provide a high level of bistability. What do you mean by 'high level'? Could you clarify or quantify this?

7. Why did you apply a different duration of input current in Fig. 1 (almost the whole simulation) than in Fig. 2 (only 100 ms)? Are these two scenarios comparable? Please, clarify.

8. Fig. 2A, B: The y-axis is fully labelled, but the x-axis is not. Please harmonise it. Again, I don't think it is necessary to show the full 120 s simulation. The lowest voltage is around -80 mV. There is no need to have a tick at -150 mV (same for Fig. 2C and 2F).

9. For the bistability windows, I would create a summary table showing I1, I2 and delta I for each scenario. This will help to understand how it changes with different modifications.

10. How many simulations did you run for the curves in Figures 2B, 2C, 2E and 2F? How did you calculate these curves? Please, clarify. Again, mark the simulated values.

11. Lines 151–152: Could you quantify or support your quotation in some way, because in my opinion the effect of KM on the bistability window is minimal?

12. Fig. 3: What step did you use to change the conductance? For example, you started with gKM=0 and increased it in steps to gKM=0.3. It is useful information for evaluating the sampling and the number of simulations you run. In addition, it would be useful to see how I1 changed for the different pCa+gKM and pCa+gKir combinations.

13. Fig. 6: More ticks are needed on the y-axis and x-axis in all subplots. I would either add ticks for 0 mV or use denser ticking (for example, 20 mV).

14. Fig. 7: I would add the exact values of I1 and I2 to all subplots for easier understanding.

15. Fig. 8: Use the exact values of I1. Add more ticks to the y-axis (A–D). Plotting only the most important part of the voltage traces would be more informative than plotting the full simulation as is currently done. Does the dashed line represent I1? If so, this should be noted in the figure caption.

16. Line 365: Could you clarify what you mean by 'after a significant increase in pCaL'?

Reviewer #3: Summary and general comments

The article illustrates the effect of L-type calcium channels in

combination with different potassium channels on membrane voltage

bistability in a computational model neuron. It agues for a trade off

between membrane bistability and physiological resting states. In

particular, the authors report that Kir and KM potassium channels have

disparate effects on bistability, while stating the exact mechanisms

remain unclear.

The article, does not provide any mechanistic insight into the observed

phenomena. It is also not clear how idiosyncratic the observed effects

are to the specific model in use. The article does not provide a

rigorous sensitivity analysis of the results, nor analytical arguments.

Consequently we would suggest the manuscript is more suited for PLOS

ONE.

Specific comments

The main conclusion of Fig 1. is that the addition of L-type calcium

channels introduces bistability into a neuron model. I would be cleaner

if the results, particularly the f-I curve and the resting voltages in

panel C would also contain the date from the model without L-type

calcium channel for comparison. After all, the model could already show

bistability to begin with.

Could the authors provide a brief description of the mechanism behind

the strong resting voltage reduction of the Calcium channels.

From the writing in this first results section it is not clear how

idiosyncratic to the specific model the effects are. Furthermore, other

manipulations of ion channel conductances can also induce bistability.

For example altering only the conductances if I_(Na) and I_(KDR) would

for some values also lead to bistability.

Could the authors supply citations for the statement that “in numerous,

instances, the increase in the conductance of calcium channels is

assumed to be coupled with an increase … of … potassium channels”?

All the x-axis of Fig. 1C and Fig. 2B, E are different in scaling and

thus range Δf are hard to compare.

On the one hand, the authors mention that in the presence of M-type

potassium channels the model fails to maintain spiking and bistability

can not be maintained, on the other hand panel B shows a bistable f-I

curve. In the text it is stated that range “appear narrower” after the

addition of M-type potassium channels. How is this then defined and can

this please be quantified.

What is the mechanism of the spike failure? M-type potassium currents

typically lead to spike frequency adaptation and can terminate spiking.

They may however be still bistable in the un-adapted state.

For the reader to appreciate the different properties it would be useful

to plot the steady state I-V relations and dynamic currents during the

AP of Kir vs M-type as well as their time constants.

Fig. 3 seems to show that the depolarising CaL channel hyperpolarises

the resting potential, while the repolarising potassium channels

depolarise the resting potential. Is this due to the model relying on

the Goldman-Hodgekin-Katz formalism?

**********

**Have the authors made all data and (if applicable) computational code underlying the findings in their manuscript fully available?**

The PLOS Data policy requires authors to make all data and code underlying the findings described in their manuscript fully available without restriction, with rare exception (please refer to the Data Availability Statement in the manuscript PDF file). The data and code should be provided as part of the manuscript or its supporting information, or deposited to a public repository. For example, in addition to summary statistics, the data points behind means, medians and variance measures should be available. If there are restrictions on publicly sharing data or code —e.g. participant privacy or use of data from a third party—those must be specified.

Reviewer #1: Yes

Reviewer #2: Yes

Reviewer #3: Yes

**********

PLOS authors have the option to publish the peer review history of their article (what does this mean?). If published, this will include your full peer review and any attached files.

If you choose “no”, your identity will remain anonymous but your review may still be made public.

**Do you want your identity to be public for this peer review?**  For information about this choice, including consent withdrawal, please see our Privacy Policy.

Reviewer #1: No

Reviewer #2: No

Reviewer #3: No

[NOTE: If reviewer comments were submitted as an attachment file, they will be attached to this email and accessible via the submission site. Please log into your account, locate the manuscript record, and check for the action link "View Attachments". If this link does not appear, there are no attachment files.]

**Figure resubmission:**

While revising your submission, we strongly recommend that you use PLOS’s NAAS tool (https://ngplosjournals.pagemajik.ai/artanalysis) to test your figure files. NAAS can convert your figure files to the TIFF file type and meet basic requirements (such as print size, resolution), or provide you with a report on issues that do not meet our requirements and that NAAS cannot fix.

After uploading your figures to PLOS’s NAAS tool - https://ngplosjournals.pagemajik.ai/artanalysis, NAAS will process the files provided and display the results in the "Uploaded Files" section of the page as the processing is complete. If the uploaded figures meet our requirements (or NAAS is able to fix the files to meet our requirements), the figure will be marked as "fixed" above. If NAAS is unable to fix the files, a red "failed" label will appear above. When NAAS has confirmed that the figure files meet our requirements, please download the file via the download option, and include these NAAS processed figure files when submitting your revised manuscript.

**Reproducibility:**

To enhance the reproducibility of your results, we recommend that authors of applicable studies deposit laboratory protocols in protocols.io, where a protocol can be assigned its own identifier (DOI) such that it can be cited independently in the future. Additionally, PLOS ONE offers an option to publish peer-reviewed clinical study protocols. Read more information on sharing protocols at https://plos.org/protocols?utm_medium=editorial-email&utm_source=authorletters&utm_campaign=protocols

Attachment Submitted filename: Review.pdf

# Author comment: Author response to Decision Letter 1

**DOI:** 10.1371/journal.pcbi.1014549.r002

3 Dec 2025

Attachment Submitted filename: response_to_reviewers.pdf

# Peer review: Decision Letter 1

**DOI:** 10.1371/journal.pcbi.1014549.r003

**Authors:** Hermann Cuntz, Marieke Karlijn van Vugt

19 Mar 2026

PCOMPBIOL-D-25-01035R1

Inward rectifier potassium channels interact with calcium channels to promote robust and physiological bistability

PLOS Computational Biology

Dear Dr. De Worm,

Thank you for submitting your manuscript to PLOS Computational Biology. After careful consideration, we feel that it has merit but does not fully meet PLOS Computational Biology's publication criteria as it currently stands. Therefore, we invite you to submit a revised version of the manuscript that addresses the points raised during the review process.

Please submit your revised manuscript by May 19 2026 11:59PM. If you will need more time than this to complete your revisions, please reply to this message or contact the journal office at ploscompbiol@plos.org. When you're ready to submit your revision, log on to https://www.editorialmanager.com/pcompbiol/ and select the 'Submissions Needing Revision' folder to locate your manuscript file.

Please include the following items when submitting your revised manuscript:

* A letter that responds to each point raised by the editor and reviewer(s). You should upload this letter as a separate file labeled 'Response to Reviewers'. This file does not need to include responses to formatting updates and technical items listed in the 'Journal Requirements' section below.

* A marked-up copy of your manuscript that highlights changes made to the original version. You should upload this as a separate file labeled 'Revised Manuscript with Track Changes'.

* An unmarked version of your revised paper without tracked changes. You should upload this as a separate file labeled 'Manuscript'.

If you would like to make changes to your financial disclosure, competing interests statement, or data availability statement, please make these updates within the submission form at the time of resubmission. Guidelines for resubmitting your figure files are available below the reviewer comments at the end of this letter

We look forward to receiving your revised manuscript.

Kind regards,

Hermann Cuntz

Academic Editor

PLOS Computational Biology

Marieke van Vugt

Section Editor

PLOS Computational Biology

**Additional Editor Comments (if provided):**

Sorry for the delay. There have been major technical problems with the editorial manager. They are still unsolved. Please address the important concerns by Reviewer 1. If you do not see the attached pdf (I don't) please let the PLoS staff know.

**Journal Requirements:**

Please amend your detailed Financial Disclosure statement. This is published with the article. It must therefore be completed in full sentences and contain the exact wording you wish to be published.

- State what role the funders took in the study. If the funders had no role in your study, please state: "The funders had no role in study design, data collection and analysis, decision to publish, or preparation of the manuscript.".

If you did not receive any funding for this study, please simply state: u201cThe authors received no specific funding for this work.u201d

**Reviewers' comments:**

Reviewer's Responses to Questions

**Comments to the Authors:**

**Please note here if the review is uploaded as an attachment.**

Reviewer #1: See attatchment.

Reviewer #2: I have carefully read the revised manuscript. The authors have adequately addressed all of my previous questions and have satisfactorily clarified the issues I raised. The figures are now clearer and more readable, the overall text has improved, and the discussion has been strengthened. I have no further comments and recommend the manuscript for acceptance.

The review is not uploaded as an attachment.

**********

**Have the authors made all data and (if applicable) computational code underlying the findings in their manuscript fully available?**

The PLOS Data policy requires authors to make all data and code underlying the findings described in their manuscript fully available without restriction, with rare exception (please refer to the Data Availability Statement in the manuscript PDF file). The data and code should be provided as part of the manuscript or its supporting information, or deposited to a public repository. For example, in addition to summary statistics, the data points behind means, medians and variance measures should be available. If there are restrictions on publicly sharing data or code —e.g. participant privacy or use of data from a third party—those must be specified.

Reviewer #1: None

Reviewer #2: Yes

**********

PLOS authors have the option to publish the peer review history of their article (what does this mean?). If published, this will include your full peer review and any attached files.

If you choose “no”, your identity will remain anonymous but your review may still be made public.

**Do you want your identity to be public for this peer review?**  For information about this choice, including consent withdrawal, please see our Privacy Policy.

Reviewer #1: No

Reviewer #2: No

[NOTE: If reviewer comments were submitted as an attachment file, they will be attached to this email and accessible via the submission site. Please log into your account, locate the manuscript record, and check for the action link "View Attachments". If this link does not appear, there are no attachment files.]

**Figure resubmission:**

While revising your submission, we strongly recommend that you use PLOS’s NAAS tool (https://ngplosjournals.pagemajik.ai/artanalysis) to test your figure files. NAAS can convert your figure files to the TIFF file type and meet basic requirements (such as print size, resolution), or provide you with a report on issues that do not meet our requirements and that NAAS cannot fix.

After uploading your figures to PLOS’s NAAS tool - https://ngplosjournals.pagemajik.ai/artanalysis, NAAS will process the files provided and display the results in the "Uploaded Files" section of the page as the processing is complete. If the uploaded figures meet our requirements (or NAAS is able to fix the files to meet our requirements), the figure will be marked as "fixed" above. If NAAS is unable to fix the files, a red "failed" label will appear above. When NAAS has confirmed that the figure files meet our requirements, please download the file via the download option, and include these NAAS processed figure files when submitting your revised manuscript.

**Reproducibility:**

To enhance the reproducibility of your results, we recommend that authors of applicable studies deposit laboratory protocols in protocols.io, where a protocol can be assigned its own identifier (DOI) such that it can be cited independently in the future. Additionally, PLOS ONE offers an option to publish peer-reviewed clinical study protocols. Read more information on sharing protocols at https://plos.org/protocols?utm_medium=editorial-email&utm_source=authorletters&utm_campaign=protocols

Attachment Submitted filename: Review 2.1-1.pdf

# Author comment: Author response to Decision Letter 2

**DOI:** 10.1371/journal.pcbi.1014549.r004

21 May 2026

Attachment Submitted filename: response_to_reviewers_auresp_2.pdf

# Peer review: Decision Letter 2

**DOI:** 10.1371/journal.pcbi.1014549.r005

**Authors:** Hermann Cuntz, Marieke Karlijn van Vugt

8 Jul 2026

Dear Ms De Worm,

We are pleased to inform you that your manuscript 'Inward rectifier potassium channels interact with calcium channels to promote robust and physiological bistability' has been provisionally accepted for publication in PLOS Computational Biology.

Before your manuscript can be formally accepted you will need to complete some formatting changes, which you will receive in a follow up email. A member of our team will be in touch with a set of requests.

Please note that your manuscript will not be scheduled for publication until you have made the required changes, so a swift response is appreciated.

IMPORTANT: The editorial review process is now complete. PLOS will only permit corrections to spelling, formatting or significant scientific errors from this point onwards. Requests for major changes, or any which affect the scientific understanding of your work, will cause delays to the publication date of your manuscript.

Should you, your institution's press office or the journal office choose to press release your paper, you will automatically be opted out of early publication. We ask that you notify us now if you or your institution is planning to press release the article. All press must be co-ordinated with PLOS.

Thank you again for supporting Open Access publishing; we are looking forward to publishing your work in PLOS Computational Biology.

Best regards,

Hermann Cuntz

Academic Editor

PLOS Computational Biology

Marieke van Vugt

Section Editor

PLOS Computational Biology

***********************************************************

Reviewer's Responses to Questions

**Comments to the Authors:**

**Please note here if the review is uploaded as an attachment.**

Reviewer #1: Thanks for this thorough revision. All my earlier points have been addressed and I recommend acceptance of this manuscript.

**********

**Have the authors made all data and (if applicable) computational code underlying the findings in their manuscript fully available?**

The PLOS Data policy requires authors to make all data and code underlying the findings described in their manuscript fully available without restriction, with rare exception (please refer to the Data Availability Statement in the manuscript PDF file). The data and code should be provided as part of the manuscript or its supporting information, or deposited to a public repository. For example, in addition to summary statistics, the data points behind means, medians and variance measures should be available. If there are restrictions on publicly sharing data or code —e.g. participant privacy or use of data from a third party—those must be specified.

Reviewer #1: Yes

**********

PLOS authors have the option to publish the peer review history of their article (what does this mean?). If published, this will include your full peer review and any attached files.

If you choose “no”, your identity will remain anonymous but your review may still be made public.

**Do you want your identity to be public for this peer review?**  For information about this choice, including consent withdrawal, please see our Privacy Policy.

Reviewer #1: No

# Editor report: Acceptance letter

**DOI:** 10.1371/journal.pcbi.1014549.r006

**Authors:** Hermann Cuntz, Marieke Karlijn van Vugt

PCOMPBIOL-D-25-01035R2

Inward rectifier potassium channels interact with calcium channels to promote robust and physiological bistability

Dear Dr De Worm,

I am pleased to inform you that your manuscript has been formally accepted for publication in PLOS Computational Biology. Your manuscript is now with our production department and you will be notified of the publication date in due course.

The corresponding author will soon be receiving a typeset proof for review, to ensure errors have not been introduced during production. Please review the PDF proof of your manuscript carefully, as this is the last chance to correct any errors. Please note that major changes, or those which affect the scientific understanding of the work, will likely cause delays to the publication date of your manuscript.

Soon after your final files are uploaded, unless you have opted out, the early version of your manuscript will be published online. The date of the early version will be your article's publication date. The final article will be published to the same URL, and all versions of the paper will be accessible to readers.

For Research, Software, and Methods articles, you will receive an invoice from PLOS for your publication fee after your manuscript has reached the completed accept phase. If you receive an email requesting payment before acceptance or for any other service, this may be a phishing scheme. Learn how to identify phishing emails and protect your accounts at https://explore.plos.org/phishing.

Thank you again for supporting PLOS Computational Biology and open-access publishing. We are looking forward to publishing your work!

With kind regards,

Janani Seenivasan

PLOS Computational Biology | Carlyle House, Carlyle Road, Cambridge CB4 3DN | United Kingdom ploscompbiol@plos.org | Phone +44 (0) 1223-442824 | ploscompbiol.org | @PLOSCompBiol
